# Structure predictions and functional insights into Amidase_3 domain containing *N*-acetylmuramyl-L-alanine amidases from *Deinococcus indicus* DR1

**DOI:** 10.1186/s12866-024-03225-4

**Published:** 2024-03-26

**Authors:** Malvika Modi, Menaka Thambiraja, Archana Cherukat, Ragothaman M Yennamalli, Richa Priyadarshini

**Affiliations:** 1 Department of Life Sciences, School of Natural Sciences, Shiv Nadar Institution of Eminence, Gautam Buddha Nagar, Uttar Pradesh 201314 India; 2 Department of Bioinformatics, School of Chemical and Biotechnology, SASTRA Deemed to be University, Thanjavur, Tamil Nadu 613401 India; 3 Department of Biology, Graduate School of Arts and Sciences, Wake Forest University, 1834 Wake Forest Rd, Winston-Salem, USA

## Abstract

**Background:**

*N*-acetylmuramyl-L-alanine amidases are cell wall modifying enzymes that cleave the amide bond between the sugar residues and stem peptide in peptidoglycan. Amidases play a vital role in septal cell wall cleavage and help separate daughter cells during cell division. Most amidases are zinc metalloenzymes, and *E. coli* cells lacking amidases grow as chains with daughter cells attached to each other. In this study, we have characterized two amidase enzymes from *Deinococcus indicus* DR1. *D. indicus* DR1 is known for its high arsenic tolerance and unique cell envelope. However, details of their cell wall biogenesis remain largely unexplored.

**Results:**

We have characterized two amidases Ami1_*Di*_ and Ami2_*Di*_ from *D. indicus* DR1. Both Ami1_*Di*_ and Ami2_*Di*_ suppress cell separation defects in *E. coli* amidase mutants, suggesting that these enzymes are able to cleave septal cell wall. Ami1_*Di*_ and Ami2_*Di*_ proteins possess the Amidase_3 catalytic domain with conserved –GHGG- motif and Zn^2+^ binding sites. Zn^2+^- binding in Ami1_*Di*_ is crucial for amidase activity. AlphaFold2 structures of both Ami1_*Di*_ and Ami2_*Di*_ were predicted, and Ami1_*Di*_ was a closer homolog to AmiA of *E. coli*.

**Conclusion:**

Our results indicate that Ami1_*Di*_ and Ami2_*Di*_ enzymes can cleave peptidoglycan, and structural prediction studies revealed insights into the activity and regulation of these enzymes in *D. indicus* DR1.

**Supplementary Information:**

The online version contains supplementary material available at 10.1186/s12866-024-03225-4.

## Introduction

Bacterial cells are surrounded by a peptidoglycan (PG) cell wall comprising polysaccharide strands connected by cross-linked peptides [1, 2]. The cell wall protects from osmotic lysis and maintains bacterial cell shape [3, 4]. The PG layer is constantly remodeled during growth and division by the coordinated action of PG synthases known as penicillin-binding proteins (PBPs) and PG hydrolases [4, 5]. Enzymes that degrade cell wall are collectedly known as PG-modifying enzymes [6–9] and comprises of *N*-acetylmuramyl-L-alanine amidases (NALAA), lytic transglycosylases, endopeptidases, carboxypeptidases, and *N-*acetylglucosaminidases [6–9]. Abnormal regulation of PG remodeling machinery can lead to cell lysis or aberrant cell division [2, 10]. During bacterial cell division, new PG material is laid down, followed by cytokinesis and septal PG hydrolysis to separate the newly formed daughter cells [11–15]. PG amidases play the most significant role in cell division by mediating cell wall splitting and daughter cell separation [15–17].

PG Amidases belong to the zinc metalloenzymes group and break the amide bond between MurNAc and the stem peptide. The catalytic domains of amidases are grouped into three categories – amidase_2 (NALAA-2; IPR002502), amidase_3 (NALAA-3; IPR002508), and amidase_5 (NALAA-5; IPR008044) [18]. The genome of *E. coli* encodes three periplasmic *N*-acetylmuramyl-L-alanine amidases – AmiA\B\C, which play a redundant role in bacterial cell separation [15, 19–21]. Amidases are recruited to the divisome [22, 23], and the loss of two or more amidases causes defects in septal PG cleavage, forcing daughter cells to grow attached in chains [15, 17].

The catalytic activity of PG amidases is modulated by interaction between other cell wall hydrolases [15, 16]. In *E. coli*, PG amidases activators comprise EnvC [11, 24] and NlpD [11], which contain degenerate lysostaphin-like metalloprotease (dLytM) domain of the peptidase_M23 family [25, 26]. EnvC regulates the activation of AmiA and AmiB, whereas NlpD governs the activation of AmiC [11, 26–28]. In contrast, in *Vibrio cholerae*, both NlpD and EnvC contribute to activating single amidase AmiB [29]. Unlike *E. coli*, *Caulobacter crescentus* harbors only one amidase AmiC, essential for cell viability [30–32]. In *Neisseria gonorrhoeae*, an obligate human pathogen, single autolysin AmiC is critical for proper cell separation [33]. The *Chlamydiaceae* family lacks a functional cell wall but possesses a bifunctional enzyme - AmiA, with both amidase and carboxypeptidase activities [34].

In this study, we characterized two amidases from *Deinococcus indicus* DR1, namely Ami1_*Di*_ and Ami2_*Di*_. *D. indicus* DR1 belongs to the Deinococcaceae family and is a rod-shaped, red-pigmented bacterium majorly known for high arsenic tolerance [35]. Our results indicate that both Ami1_*Di*_ and Ami2_*Di*_ from *D. indicus* are able to suppress cell separation defects in *E. coli* amidase mutants. Computational modeling revealed that both proteins possess the Amidase_3 catalytic domain with conserved –GHGG- motif and Zn^2+^-binding sites. Structures of both Ami1_*Di*_ and Ami2_*Di*_ were predicted by AlphaFold2. Structural similarity revealed Ami1_*Di*_ being a closer homolog to AmiA of *E. coli* and may follow the same amidase/activator model. Our study is the first step in characterizing amidases from an extremophile *D. indicus* and can help uncover their role in maintaining complex cell envelopes.

## Materials and methods

### Strains, media, and growth conditions

*D. indicus* DR-1 cells were grown in PYE (peptone yeast extract) [36] medium supplemented with 1 mM MgSO_4_ and 1 mM CaCl_2_ at 30 °C. *E. coli* DH5α cells were used for cloning, and BL21 (DE3) was used for protein induction and purification. For plasmid selection, *E. coli* cells were grown in LB (Luria Bertani) medium at 37 °C with kanamycin antibiotic (50 µg/mL). For induction of genes encoded under the control of L-arabinose or lactose inducible promoter, cells were grown in the presence of 0.4% L-arabinose or 0.5 µM IPTG (isopropyl-β-d-1-thiogalactopyranoside). The strains and plasmids used are mentioned in Table 1. Antibiotics used in this study were purchased from Sigma Aldrich (Milwaukee, U.S.A), and media were purchased from Himedia (Mumbai, India).

**Table 1:** Strains and plasmids used in this study

	Relevant genotype and description	Sources/References
Plasmids		
pBAD18	Kan^R^, DH5α containing empty pBAD18	[68]
pET28b	protein expression vector, Kan^R^	Laboratory strain collection
pMD01	pBAD18 carrying *ami1*_*Di*_, Kan^R^	This work
pMD02	pBAD18 carrying *ami1*_*Di*_^(1–155a)^, Kan^R^	This work
pMD03	pBAD18 carrying *ami1*_*Di*_^(H161A),^ Kan^R^	This work
pMD04	pBAD18 carrying a*mi2*_*Di*_, Kan^R^	This work
pMD05	pET28b carrying *ami1*_*Di*_, Kan^R^	This work
pMD06	pET28b carrying *ami2*_*Di*_, Kan^R^	This work
Strain		
*D. indicus*		
DR1	Wild-type	[35]
*E. coli*		
DH5α	*Φ80 ΔlacZΔM15 Δ(lacZYA-argF)U169 deoR recA1 endA hsdR17(rk-,mk+) phoA supE44 thi-1 gyrA96 relA1*	Laboratory strain collection
BL21 (DE3)	*F*^*−*^*ompT hsdS*_*B*_*(r*_*B*_^*–*^, *m*_*B*_^*−*^*) gal dcm (DE3) pLysS(Cam*^*R*^*)*	Laboratory strain collection
MG1655	*K-12 F* ^*−*^ *λ* ^*−*^ *ilvG* ^*−*^ *rfb-50 rph-1*	Laboratory strain collection
RP21	MG1655 *ΔamiA::frt ΔamiC::frt /*pBAD18	[30]
RP101	MG1655 *ΔamiA::frt ΔamiC::frt /*pMD01	This work
RP102	MG1655 *ΔamiA::frt ΔamiC::frt /*pMD02	This work
RP103	MG1655 *ΔamiA::frt ΔamiC::frt /*pMD03	This work
RP104	MG1655 *ΔamiA::frt ΔamiC::frt /*pMD04	This work
RP105	BL21(DE3)*/*pMD05	This work
RP106	*BL21(DE3)/*pMD06	This work

### Microscopy

Cells were collected at different time points and immobilized on 1% 1 X PBS agarose padded slides and were subjected to phase contrast microscopy using a Nikon Eclipse Ts2R microscope (Nikon, Japan) attached with a Nikon DS-Fi3 camera equipped with Nikon Plan Fluor 100X oil Ph3 objective. Time-lapse imaging of live cells harvested at mid-exponential phase (OD_600_ ∼ 0.4) was performed on LB 0.7% agarose padded slides supplemented with or without 0.4% L-arabinose using a Nikon Eclipse Ti microscope (Nikon, Japan) with Nikon DS-U3 camera and Plan Apo 100 X oil objective. Image processing was performed with ImageJ [37] and Adobe Photoshop CS6 (Adobe Inc. U.S.A).

### Complementation assay

*D. indicus* putative amidase *ami1*_*Di*_ and *ami2*_*Di*_ was cloned into the pBAD18 vector under the control of L-arabinose promoter and transformed into *E. coli ∆amiAC* (RP21) competent cells resulting in the formation of constructs *E. coli ∆amiAC*/pBAD*ami1*_*Di*_ (RP101) and *E. coli∆amiAC/*pBAD*ami2*_*Di*_ (RP104). To check for complementation, cells were serially diluted at a ratio of 1:100, induced with 0.4% L-arabinose at OD_600_ ∼ 0.2, and subjected to phase contrast imaging. *E.coli ∆amiAC/*pBAD18 (empty vector) induced with 0.4% L-arabinose was used as a control. Cells were counted for the number of singlets, doublets, triplets, and chains using ImageJ [37].

### Site-directed mutagenesis

Multiple sequence alignment using ClustalW [38] identified conserved active site residue between *E. coli* AmiC, *C. crescentus* AmiC, and *D. indicus* Ami1 sequences. Active site mutant was generated using the QuickChange Method (Stratagene, La Jolla, CA) [39]. Point mutation at position 161 was created by replacing Histidine with Alanine. The parental plasmids were digested with DpnI, and mutant plasmids were used to transform *E. coli* DH5α with selection on LB + kanamycin plates. Active site mutants were further confirmed by Sanger sequencing.

### Construction of the recombinant his-tag fusion proteins

The 1.17-kb gene encoding Ami1_*D.i*_ and 1.7-kb encoding Ami2_*D.i*_ were tagged with 6xHis sequence at the C-terminus using primers mentioned in supplementary Table 2. PCR purified fragments of Ami1_*Di*_ and Ami2_*Di*_ were digested with EcoRI and HindIII restriction enzymes and ligated within pET28b vector. After isolation, recombinant plasmids were confirmed by Sanger sequencing and introduced into *E. coli* BL21 (DE3) for protein production and purification.

### Protein purification

Ami1_*Di*_ and Ami2_*Di*_ were purified using cobalt-based immobilized metal ion affinity chromatography (IMAC). BL21 (DE3)/pET28b*ami1*_*Di*_ (RP105) and BL21 (DE3)/pET28b *ami2*_*Di*_ (RP106) were grown at 37 °C in 1 L Terrific Broth (TB) media supplemented with kanamycin (50 µg/mL). At OD_600_ ∼ 0.6, cells were induced with 0.5 mM IPTG for 3 h at 25 °C. The cells were collected and resuspended in 20 mL of resuspension buffer (10 mM Tris-HCl – pH 8.6, 200 mM NaCl, 10% glycerol, 1 mM PMSF). The cells were incubated on ice for 30 min followed by sonication using Q700CA sonicator at 30% amplitude for 6 min. Sonicated samples were centrifuged at 10,000 g for 40 min. Cell-free supernatant was incubated with cobalt resin (equilibrated with 10 mM Tris-HCl – pH 8.6, 200 mM NaCl, 10% glycerol) for 3 h at 4 °C with gentle shaking. The resin was collected and washed with 30 mL of Wash Buffer (10 mM Tris-HCl – pH 8.6, 200 mM NaCl, 10% glycerol, 20 mM imidazole). Protein was eluted with a wash buffer containing an increasing imidazole concentration (40 mM – 250 mM). Eluted fractions were bound on the buffer exchange column and collected using wash buffer without imidazole. Purified protein (Ami1_*D.i*_ and Ami2_*D.i*_) concentrations were measured using Bradford microassay [40] in 10 mM Tris-HCl Buffer (pH 8.6). SDS-PAGE followed by Coomassie staining, and Western Blot were performed.

### Western blotting analysis

Purified proteins (Ami1_*D.i*_ and Ami2_*D.i*_) were separated based on their molecular weight on SDS-PAGE gel (12% w/v) and transferred onto Polyvinylidenedifluoride (PVDF) membrane. After blocking with 5% skim milk in 1 X PBS buffer, the membrane was probed with rabbit anti-His tag monoclonal antibody (1:3000 dilution, Invitrogen, Waltham, MA, USA) overnight at 4 °C. The membrane was washed 3 times with PBST and probed with horseradish peroxidase-conjugated anti-rabbit antibodies (1:10000 dilution, Invitrogen, Waltham, MA, USA). Final blot was developed with Bio-Rad Clarity and Clarity Max ECL Western Blotting Substrates (Bio-Rad Laboratories, Inc. U.S.A) according to the manufacturer’s protocols.

### PG purification and hydrolytic activity assay

Peptidoglycan from *D. indicus* DR-1 was purified as described previously with slight modifications [41]. About 2 L of *D. indicus* was cultured in TSB medium and grown to an OD_600_ ~ 0.8. Cells were pelleted down at 12,000 g for 20 min and washed twice with distilled water (0.2 g/mL). The cell suspension was added dropwise into 8% boiling SDS with vigorous stirring. The solution was boiled for 2 h, and the lysate was allowed to cool down at room temperature overnight. Afterward, the solution was pelleted by ultracentrifugation at 120,000 g for 90 min at room temperature. The insoluble peptidoglycan obtained was washed at least eight times with distilled water to remove residual SDS (SDS concentration < 1 µg/mL). The final concentration of SDS in insoluble peptidoglycan was determined by the Methylene Blue assay [42]. Isolated PG was resuspended in 3 mL of Tris (10 mM) buffer. Remazol brilliant blue (RBB) labeled sacculi was prepared as described previously [26]. 0.2 g of insoluble peptidoglycan was resuspended in 10 mL of 0.25 M NaOH containing 20 mM RBB. The suspension was incubated overnight at 37 °C and then washed repeatedly with distilled water until the supernatant was clear. For the hydrolytic activity assay, 100 µL RBB- labeled sacculi were incubated with about 2 µg of purified protein in 100 µL of PBS buffer (10 mM Na_2_HPO_4_, 2 mM KH_2_PO_4_, 137 mM NaCl and 2.7 mM KCl, pH 7.4) and incubated at 37 °C for different time points. Lysozyme (2 µg) and protein wash buffer were used as control. Reactions were pelleted down at 16,000 g for 10 min. Hydrolyzed PG was determined spectrophotometrically at 595 nm by measuring the concentration of released RBB dye in the collected supernatant.

### Computational models of Ami1_*Di*_ and Ami2_*Di*_

The sequences of Ami1_*Di*_ (Accession id: WP_229844239) and Ami2_*Di*_ (Accession id: WP_088250252) from the *D. indicus* DR1 genome were extracted from NCBI. We employed AlphaFold2 [43] as implemented in ColabFold [44] pipeline. We searched for suitable templates and carried out three rounds of AMBER-based energy relaxation, yielding a total of five structural models. These models were subsequently ranked based on their pLDDT scores ranging from 0 to 100. A higher pLDDT score is indicative of a more reliable and high-quality structure. The structurally equivalent residues for the zinc (Zn^2+^) binding site in both amidases were identified visually using PyMOL (The PyMOL Molecular Graphics System, Version 2.0 Schrödinger, LLC). We used AmiC from *Escherichia coli* (PDB id: 4BIN) as the reference protein to identify the structurally equivalent residues in Ami1_*Di*_ and Ami2_*Di*_. Structurally similar amidase proteins from various organisms using Ami1_*Di*_ and Ami2_*Di*_ as query structures were searched using the DALI server [45] and structures having a Z-score above 10 were considered for further analysis to identify structural homology.

### Phylogenetic tree analysis

We generated phylogenetic trees using the MEGA 11 [46] tool for both Ami1_*Di*_ and Ami2_*Di*_ by comparing them with structurally similar amidase proteins identified through the DALI server, as well as AmiC from *C. crescentus*. However, we conducted two separate analyses to build phylogenetic trees, one for Ami1_*Di*_ and one for Ami2_*Di*_. The Ami1_*Di*_ analysis involved 16 amino acid sequences, consisting of 14 sequences having structural homology with Ami1_*Di*_ according to Z-score predicted by DALI server, one sequence representing Ami1_*Di*_ from *D. indicus*, and one sequence corresponding to AmiC from *C. crescentus*. Meanwhile, the Ami2_*Di*_ analysis comprised 14 amino acid sequences, including 12 sequences having structural homology to Ami2_*Di*_ from DALI, one sequence from *D. indicus* representing Ami2_*Di*_, and one sequence representing AmiC from *C. crescentus*. We utilized the ClustalW algorithm to align the protein sequences. The evolutionary history was deduced utilizing the Neighbor-Joining method [47], with a bootstrap consensus tree based on 1000 replicates to represent the evolutionary relationships among the analyzed taxa [48]. Branches representing partitions that appeared in less than 50% of the bootstrap replicates were collapsed. The percentage of replicate trees where the associated taxa clustered together during the bootstrap test (1000 replicates) is displayed adjacent to the branches. Evolutionary distances were calculated using the Jones-Taylor-Thomton (JTT) model, a matrix-based method [49], and are expressed in terms of the number of amino acid substitutions per site. Ambiguous positions were excluded for each sequence pair (pairwise deletion option). The final dataset consisted of 870 positions for Ami1_*Di*_ and 895 positions for Ami2_*Di*_, and these positions indicate the total number of aligned sites in the amino acid sequence that were used to construct the phylogenetic tree and infer the evolutionary relationship.

## Results

### Phylogenetic analysis

**Fig. 1:**
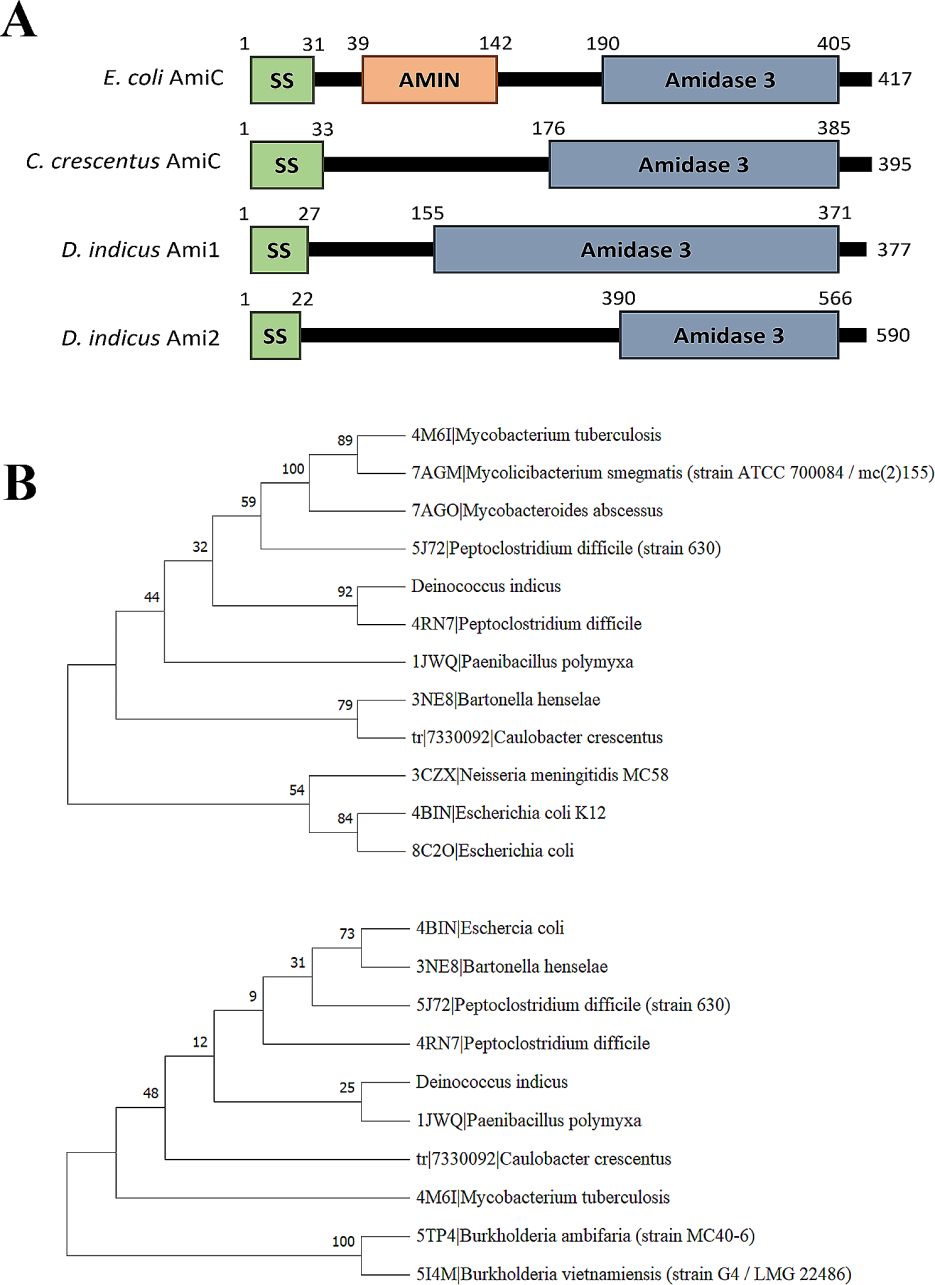
Domain architecture and phylogenetic analysis of *D. indicus* amidases. (**A**) Domain organization of *N*-acetyl-muramyl amidase of *E. coli* (AmiC), *C. crescentus* (AmiC), *D. indicus* Ami1_*Di*_ and Ami2_*Di*_ protein representing conserved Amidase_3 domain. (**B**) Phylogenetic trees. (i) Phylogenetic tree of Ami1_*Di*_ with the structural homologs having a Z-score of more than 10 from DALI. (ii) Phylogenetic tree of Ami2_*Di*_ with the structural homologs having a Z-score of more than 10 from DALI

Blast analysis revealed that *the D. indicus* genome encodes two cell wall amidases annotated here as Ami1_*Di*_ (WP_229844239) and Ami2_*Di*_ (WP_088250252). Both proteins are predicted to have approximately 30 amino acid signal peptide sequences at the N-terminus. Unlike *E. coli* AmiC [50], the AMIN domain was absent in both *D. indicus* amidase proteins (Fig. 1A), and both proteins lack conventional cell wall targeting and peptidoglycan binding domains. At the C-terminus, both proteins show high similarity to the Amidase_3 domain, indicating that they belong to the Amidase_3 family, which includes zinc-dependent enzymes (Fig. 1A). The Amidase_3 domain of Ami2_*Di*_ was found to be smaller in size (176 aa) in comparison to the Amidase_3 domain of Ami1_*Di*_.

Figure 1B shows a phylogenetic tree generated by the MEGA 11.0 program, where we found that both amidases are closely associated with the *N*-acetylmuromoyl-L-alanine amidase from two distinct organisms. Specifically, Ami1_*Di*_ demonstrated a close relationship with *Peptoclostridium difficile* (Fig. 1B-i), while Ami2_*Di*_ exhibited a close association with *Paenibacillus polymyxa* (Fig. 1B-ii). These highlight possible reasons for their functional and structural differences. Using the DALI server, we predicted a total of 14 structural homologs for Ami1_*Di*_ and 12 structural homologs for Ami2_*Di*_, all of which had a Z-score above 10, as documented in Table S1. Notably, Ami1_*Di*_ exhibited two distinct hits that were presented in Ami2_*Di*_. These unique hits in Ami1_*Di*_ included the structure of the putative *N*-acetylmuromoyl-L-alanine amidase from *Neisseria meningitidis* and the Thermosome subunit from *Methanococcoides burtonii*. The remaining hits were common to Ami1_*Di*_ and Ami2_*Di*_ with varying Z-scores (Table S1).

### Ami1_*Di*_ and Ami2_*Di*_ from *D. Indicus* are functional in *E. Coli*

**Fig. 2:**
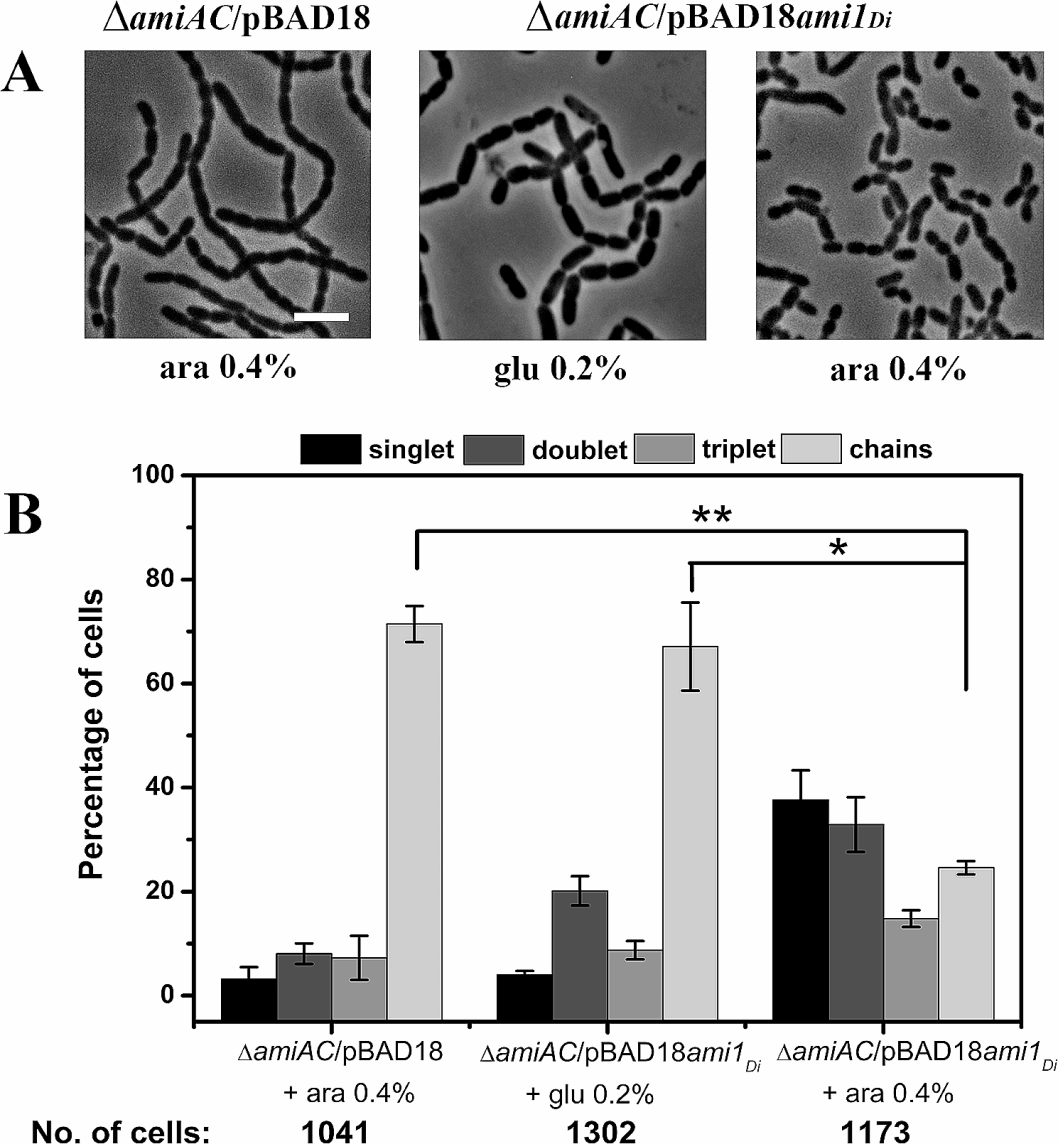
Ami1_*Di*_ can suppress cell separation defects in *E. coli* amidase mutant. (**A**) Phase contrast micrographs representing cells of strain RP21 (MG1655 *ΔamiA::frt ΔamiC::frt* /pBAD18), RP101 (MG1655 *ΔamiA::frt ΔamiC::frt* /pBAD18*ami1*_*Di*_) induced with 0.4% L-arabinose (ara). Cells were grown in LB medium at 37 °C to an OD_600_ ∼ 0.2 and induced with 0.4% L-arabinose (ara). RP101 strain grown with 0.2% glucose (glu) was used as a negative control. After 6 h of induction, cells were collected and 6 µL was immobilized on 1XPBS agarose pad and imaged. In the presence of 0.4% L-arabinose complementation of chain-forming double-amidase mutant by *ami1*_*Di*_ resulted in a reduction in the chaining phenotype. (**B**) Quantitative analysis of phase contrast micrographs of strains RP21 and RP101. Cells were counted as mentioned in Materials and Methods. RP101 shows a reduction in chains and an increment in singlets and doublets. Datasets are from three independent experiments, and error bars represent standard deviation. P value = RP21 vs. RP101 + 0.2% glu (glucose), *p* < 0.05**, RP21 vs. RP101 + 0.4% ara (L-arabinose), *p* < 0.05**. No. of cells – Total number of cells counted for each strain

To investigate the role of *D. indicus* amidases in daughter cell separation, we introduced full-length *ami1*_*Di*_ on pBAD18 plasmid into *E. coli* cells lacking both *amiA* and *amiC* (RP101). RP101 cells, when grown in the presence of L-arabinose, showed an increase in single and paired cells and a decrease in chains (Fig. 2A). In contrast, RP101 cells grown in LB medium supplemented with glucose and control cells (RP21) harboring empty pBAD vector, displayed 71% and 67% of cells attached in chains. Quantitative analysis of experiments revealed that due to the induction of Ami1_*Di*_ with L-arabinose, there was about a 40% decrease in chains, a 32% increase in singlets, a 30% increase in doublets, and a 5% increase in triplets (Fig. 2B) compared to the control cells carrying empty vector, suggesting that Ami1_*Di*_ can suppress cell separation defects in *E. coli* amidase mutants.

**Fig. 3:**
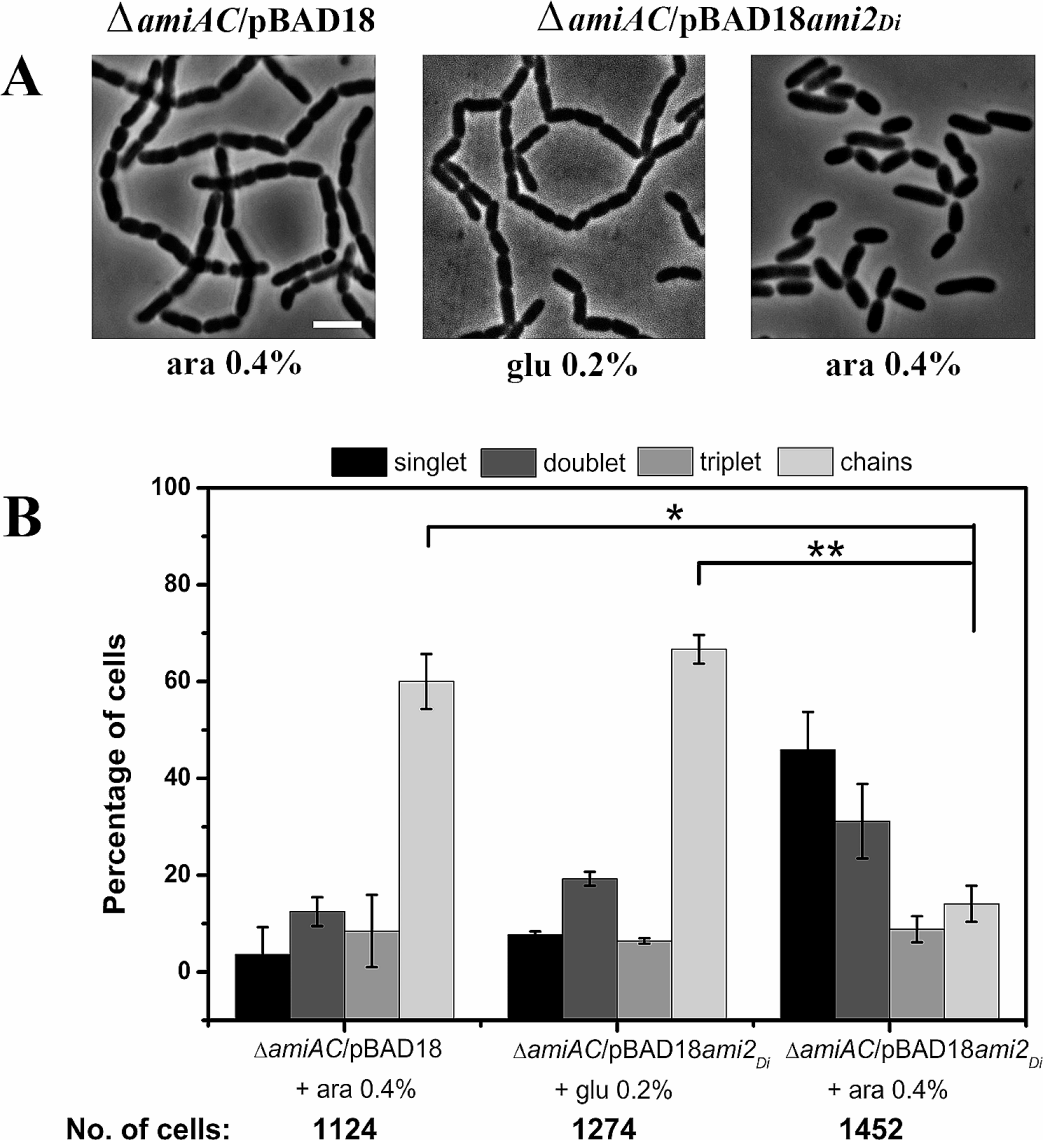
*D. indicus* Ami2_*Di*_ restores cytokinesis in *E. coli* amidase mutant. (**A**) Phase contrast micrographs representing cells of strain RP21 (MG1655 *ΔamiA::frt ΔamiC::frt* /pBAD18), RP104 (MG1655 Δ*amiA*::*frt ΔamiC*::*frt* /pBAD18*ami2*_*Di*_) induced with 0.4% L-arabinose. The complementation of *E. coli* amidase mutant by *ami2*_*Di*_ with 0.4% L-arabinose reduced chaining. (**B**) Quantitative analysis of phase contrast micrographs of strains RP21 and RP104. On induction with L-arabinose, RP104 cells show a significant reduction in chains and increment in singlets and doublets cells. Cells were counted as mentioned in Materials and Methods. Datasets are from three independent experiments, and error bars represent standard deviation. P value = RP21 vs. RP104 + 0.2% glu (glucose), *p* > 0.05*, RP21 vs. RP104 + 0.4% ara (arabinose), *p* < 0.05**. No. of cells – Total number of cells counted for each strain

Similarly, we expressed Ami2_*Di*_ under the control of L-arabinose promoter in *E. coli* cells deleted for both AmiA and AmiC (RP104). Post induction with L-arabinose, a significant decrease in the number of cells in chains was observed (Fig. 3A), indicating that Ami2_*Di*_ may have amidase activity and can suppress cell separation defects. Compared to control cells grown in glucose, where 60% of cells were present in chains, in RP104 under L-arabinose induction only about 14% of the cell population was in chains (Fig. 3B). Increase in single cells (~ 45%) and double cells (~ 31%) was also observed suggesting that cell division and cytokinesis were taking place in RP104 (Fig. 3B). Taken together our results indicate that both Ami1_*Di*_ and Ami2_*Di*_ have cell wall hydrolytic activity and may play a role in daughter cell splitting after cell division.

### Overexpression of *ami_2*_*Di*_ leads to cell lysis

**Fig. 4:**
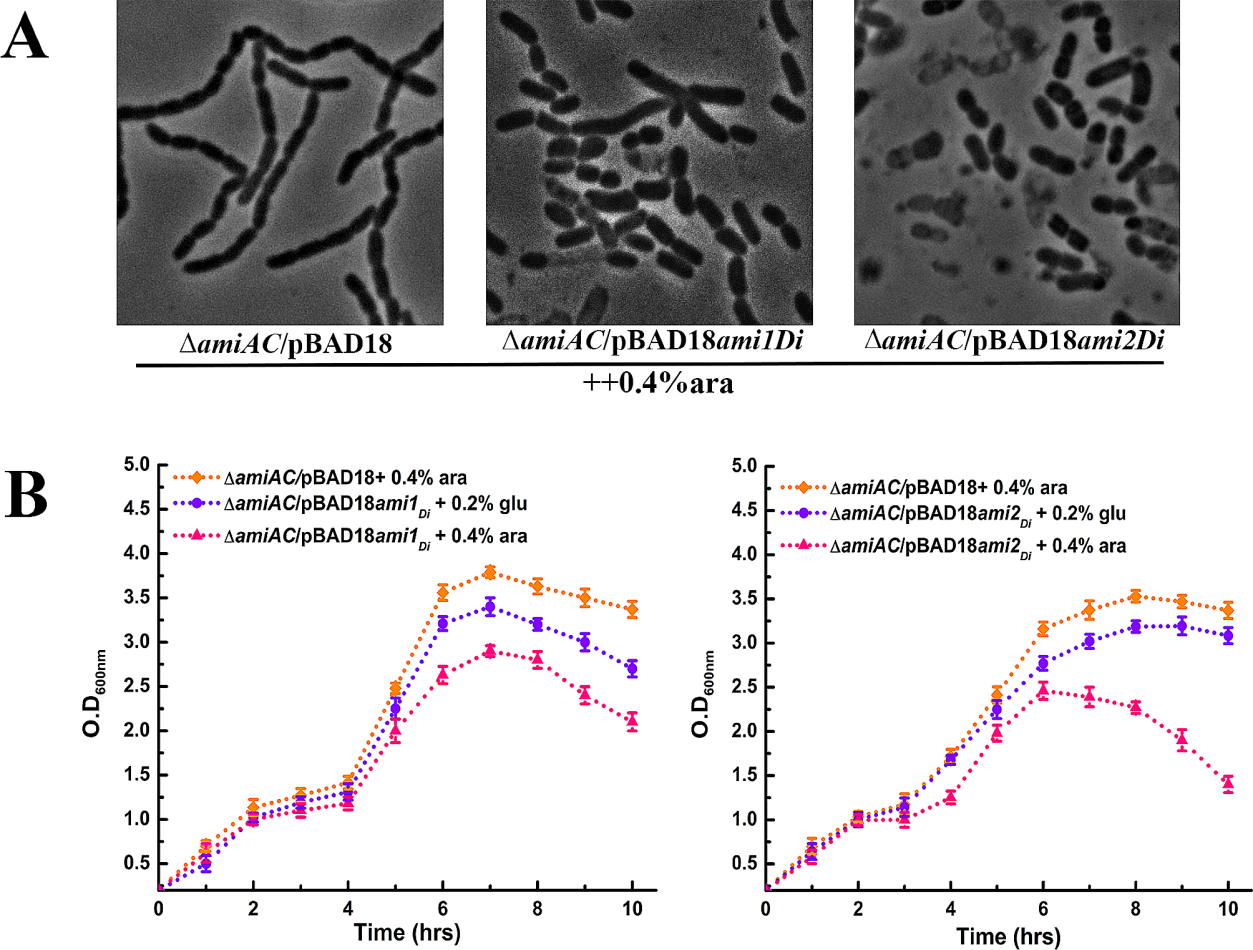
Overexpression of *ami2*_*Di*_ leads to cell lysis in *E. coli*. (**A**) Phase contrast images of strain RP101 and RP104 showing lytic activity of *ami1*_*Di*_ and *ami2*_*Di*_, respectively on overexpressing under the control of L-arabinose promoter. Strain RP21, RP101 and RP104 were grown till OD_600_ ∼ 0.2 and induced with L-arabinose for 10 h. Cells were collected, and about 6 µL sample was immobilized on 1XPBS agarose pad and imaged. Cells overexpressing *ami2*_*Di*_ (RP104) show higher lytic activity as represented by dead cells. (**B**) Growth curve assay confirming the elevated lytic activity of *ami2*_*Di*_ (RP104). Strain RP21, RP101, and RP104 were induced with L-arabinose at OD_600_ ∼ 0.1. Optical density was measured every 1 h. Cells induced with 0.2% glucose were used as the negative control. Datasets are from three biological replicates, and error bars represent standard deviation

Overexpression of *N*-acetylmuramyl-L-alanine amidases can lead to bacteriolysis, suggesting amidases to be powerful lytic enzymes [15, 51]. To examine if over activity of *D. indicus* amidases in *E. coli* would lead to cell lysis, RP101 and RP104 cells were induced with 0.4% L-arabinose. After 4 h post-induction with L-arabinose, cells overexpressing *ami1*_*Di*_ showed decreased growth (Fig. 4B) and cell lysis (Fig. 4A). However, there was a drastic reduction in cell viability (Fig. 4B) and enhanced lysis in cells overexpressing *ami2*_*Di*_ (Fig. 4A), suggesting that *ami2*_*Di*_ probably has stronger cell wall hydrolytic activity. In contrast, the control cell harboring empty pBAD plasmid did not exhibit cell lysis under similar conditions.

### Structural analysis of Ami1_Di_ and Ami2_Di_

**Fig. 5:**
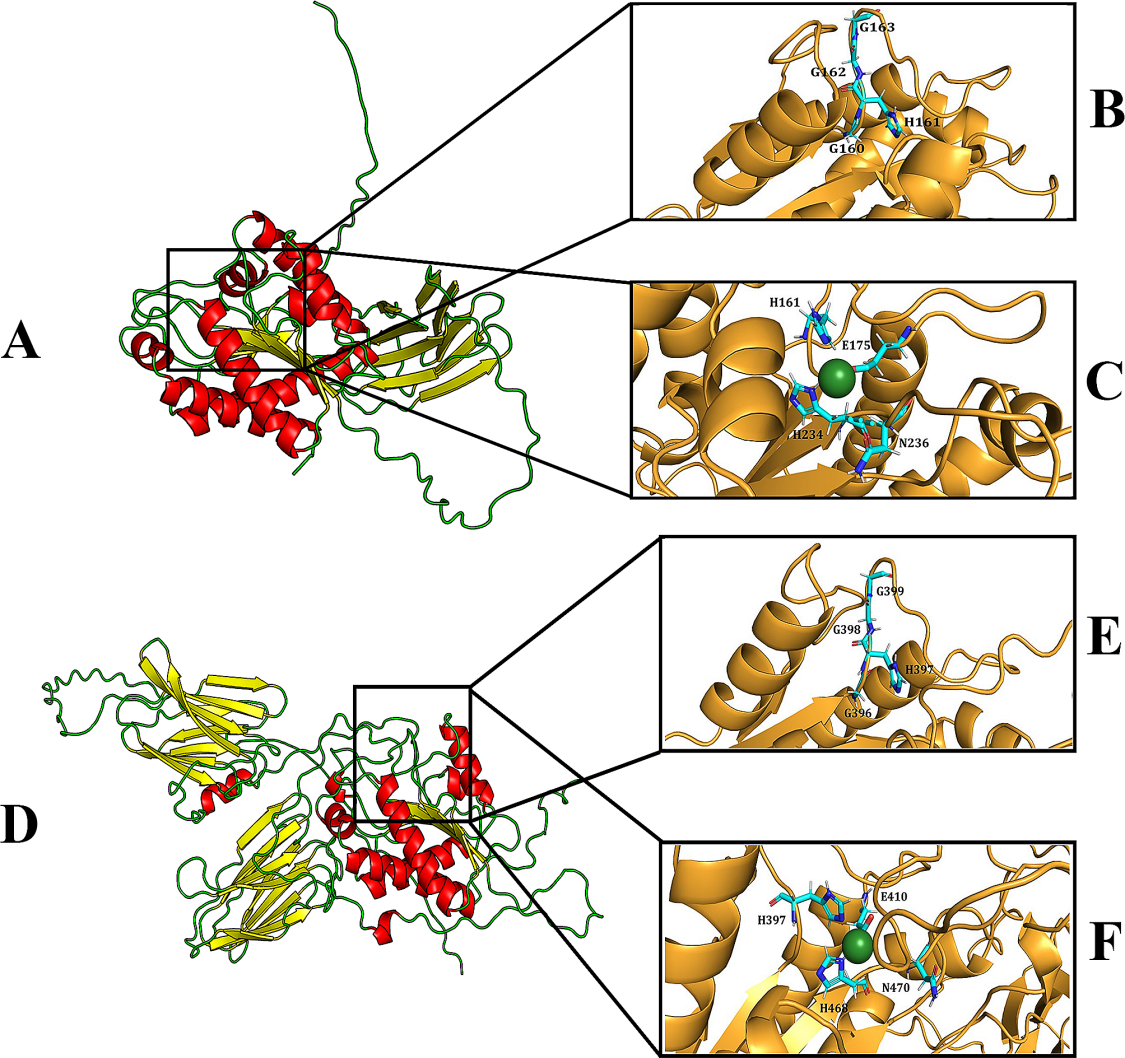
Structural analysis of Ami1_*Di*_ and Ami2_*Di*_ showing zinc binding and active site motifs. **(A**) Cartoon representation of Ami1_*Di*_, colored based on secondary structure (helix, sheet, and loop are shown in red, yellow, and green, respectively). **(B)** Close-up on the conserved active site motif of Ami1_*Di*_. The motifs are represented as sticks, where nitrogen and oxygen are shown in blue and red, respectively. **(C)** Close-up on the equivalent residue for zinc binding sites of Ami1_*Di*_. The zinc and surrounding helix and sheets are shown in green and bright orange, respectively. The chelating residues are represented as sticks (nitrogen and oxygen are shown in blue and red, respectively). **(D)** Cartoon representation of Ami2_*Di*_, colored based on secondary structure (helix, sheet, and loop are shown in red, yellow, and green, respectively). **(E)** Close-up on the conserved active site motif of Ami2_*Di*_. The motifs are represented as sticks, where nitrogen and oxygen are shown in blue and red, respectively. **(F)** Close-up on the equivalent residue for zinc binding sites of Ami2_*Di*_. The zinc and surrounding helix and sheets are shown in green and bright orange, respectively. The chelating residues are represented as sticks (nitrogen and oxygen are shown in blue and red, respectively)

AlphaFold2 predicted the structures of both Ami1_*Di*_ and Ami2_*Di*_ with reasonable accuracy, as indicated by their respective pLDDT scores of 82.7 and 79.1, along with corresponding ptm scores of 0.593 and 0.616 (Fig S1 & S2). These scores demonstrate the suitability of these predicted structures for further analysis. Ami1_*Di*_ is predicted to possess a two-domain structure, consisting of a β-sandwich N-terminal domain and an α/β C-terminal domain connected by an alanine-rich linker region (Fig. 5A). Notably, the initial 25 residues of Ami1_*Di*_ are predicted to be disordered. Ami2_*Di*_ is also a two-domain protein but exhibits significant differences compared to Ami1_*Di*_. It is predicted as a three-domain protein, featuring a β-sandwich N-terminal domain, an α/β C-terminal domain, and an additional β-sandwich domain situated between the two primary domains (Fig. 5D). Interestingly, this α/β domain is shared between both proteins. We hypothesize that it is functionally relevant as the Zn^2+^-binding region and -GHGG- motif is present in this domain. Remarkably, the α/β domain is shared among all structurally analogous amidase proteins exhibiting a Z-score above 10, as predicted by the DALI server. Specifically, Ami1_Di_ demonstrates a closer structural resemblance to the novel amidase from *Mycobacterium tuberculosis* (PDB id: 4lQ6), with a Root Mean Square Deviation (RMSD) of 2.89 Å calculated over 176 residues, compared to other amidases from various organisms. Similarly, Ami2_Di_ exhibits a higher structural similarity to the catalytic domain of *N*-acetylmuramoyl-L-alanine amidase from *Paenibacillus polymyxa* (PDB id: 1JWQ), with an RMSD of 1.84 Å calculated over 168 residues, compared to other amidases from various organisms. Similar to Ami1_*Di*_, Ami2_*Di*_ exhibits a 65-residue disordered N-terminal region. Furthermore, a structural comparison of the top-ranked models for Ami1_*Di*_ and Ami2_*Di*_, conducted using PyMOL, revealed a superimposed region shared by both proteins, potentially of significant functional relevance (Fig. S3). We identified the conserved active site motif -GHGG- in both amidases. In Ami1_*Di*_, this motif is comprised of residues G160, H161, G162, and G163 (Fig. 5B), while in Ami2_*Di*_, it is comprised of residues G396, H397, G398, and G399 (Fig. 5E). We have also identified the structurally equivalent residues for the zinc-binding sites in both Ami1_*Di*_ and Ami2_*Di*_, as depicted in Fig. 5C and F. In Ami1_*Di*_, these residues are H161, E175, H234, and N236, while in Ami2_*Di*_, they are H397, E410, H468, and N470 (Fig. 5D and F).

### Ami1_*Di*_ and Ami2_*Di*_ have peptidoglycan cleavage activity

**Fig. 6:**
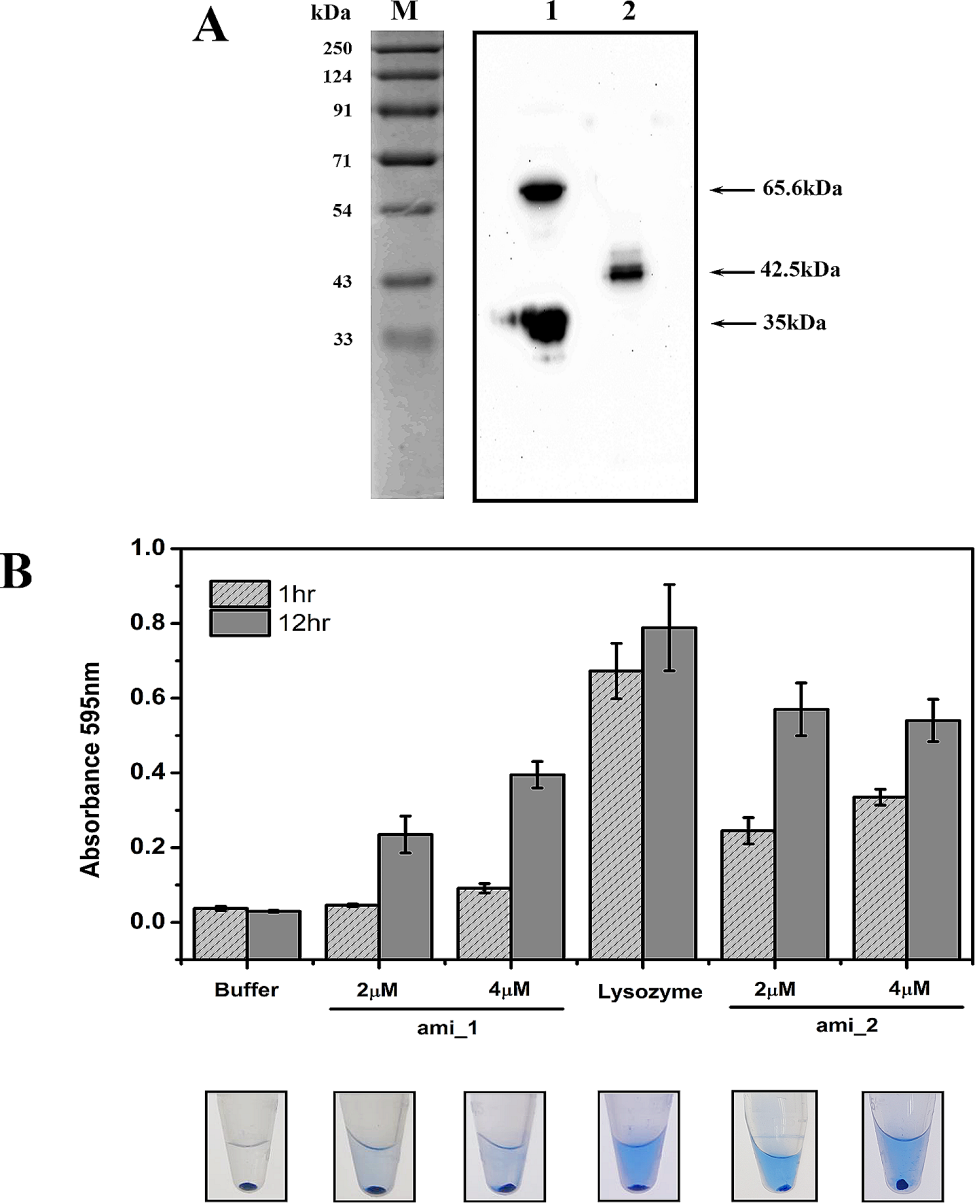
Peptidoglycan hydrolytic cleavage activity of Ami1_*Di*_ and Ami2_*Di*_. (**A**) Western blot showing purified His-tagged Ami2_*Di*_ (65.6 kDa) – Lane 1 and Ami1_*Di*_ (42.5 kDa) – Lane 2 protein heterologously produced in *E.coli* BL21(DE3) strain (RP106 & RP105). M- Protein marker. (**B**) Determination of the PG hydrolase activity of Ami1_*Di*_ and Ami2_*Di*_. Remazol brilliant blue (RBB)-labeled peptidoglycan was incubated with Ami1_*Di*_ (2 µM and 4µM) and Ami2_*Di*_ (2 µM and 4 µM), lysozyme as positive control and buffer as negative control at 37 °C. At 1 h and 12 h, the samples were pelleted down, and the absorbance of the supernatant was measured at 595 nm. The pictures below the panel show the results with an incubation of 12 h. Three biological replicate experiments were performed for each reaction, with error bars representing the standard deviation

Our experiments showed that both amidase Ami1_*Di*_ and Ami2_*Di*_ can perform PG splitting in *E. coli* cells. To investigate whether both *D. indicus* amidases can cleave *D. indicus* cell wall, we purified C-terminus His-tag variant of both proteins (Fig. 6A). We observed that the purified Ami2_*Di*_ protein showed the presence of two bands in western blot (Fig S4B), one at full length of 65.6 kDa and a smaller band at 35 kDa (Fig. 6A). Heterologous expression of *Deinococcus indicus* proteins in *E.coli* could lead to protein stability or folding issues which may have caused partial degradation of Ami2_*Di*_ protein. Peptidoglycan cleavage activity was tested by dye release assay on RBB labeled PG. Purified Ami1_*Di*_ and Ami2_*Di*_ proteins were incubated with RBB labeled PG for short (1 h) and long (12 h) - time intervals to measure peptidoglycan cleavage activity. At shorter incubation, only Ami2_*Di*_ showed significant enzymatic activity, corroborating our previous results that Ami2_*Di*_ may be a more potent PG hydrolase (Fig. 6B). Both enzymes showed increased cell wall hydrolysis at longer incubation period. Lysozyme was used as a positive control in the above experiment. At extended incubation times, PG degradation by both amidase enzymes was comparable to that obtained with lysozyme (Fig. 6B). Our results indicate that both Ami1_*Di*_ and Ami2_*Di*_ can act as PG hydrolase.

### Amidase_3 domain is essential for the lytic activity of Ami1_*Di*_

**Fig. 7:**
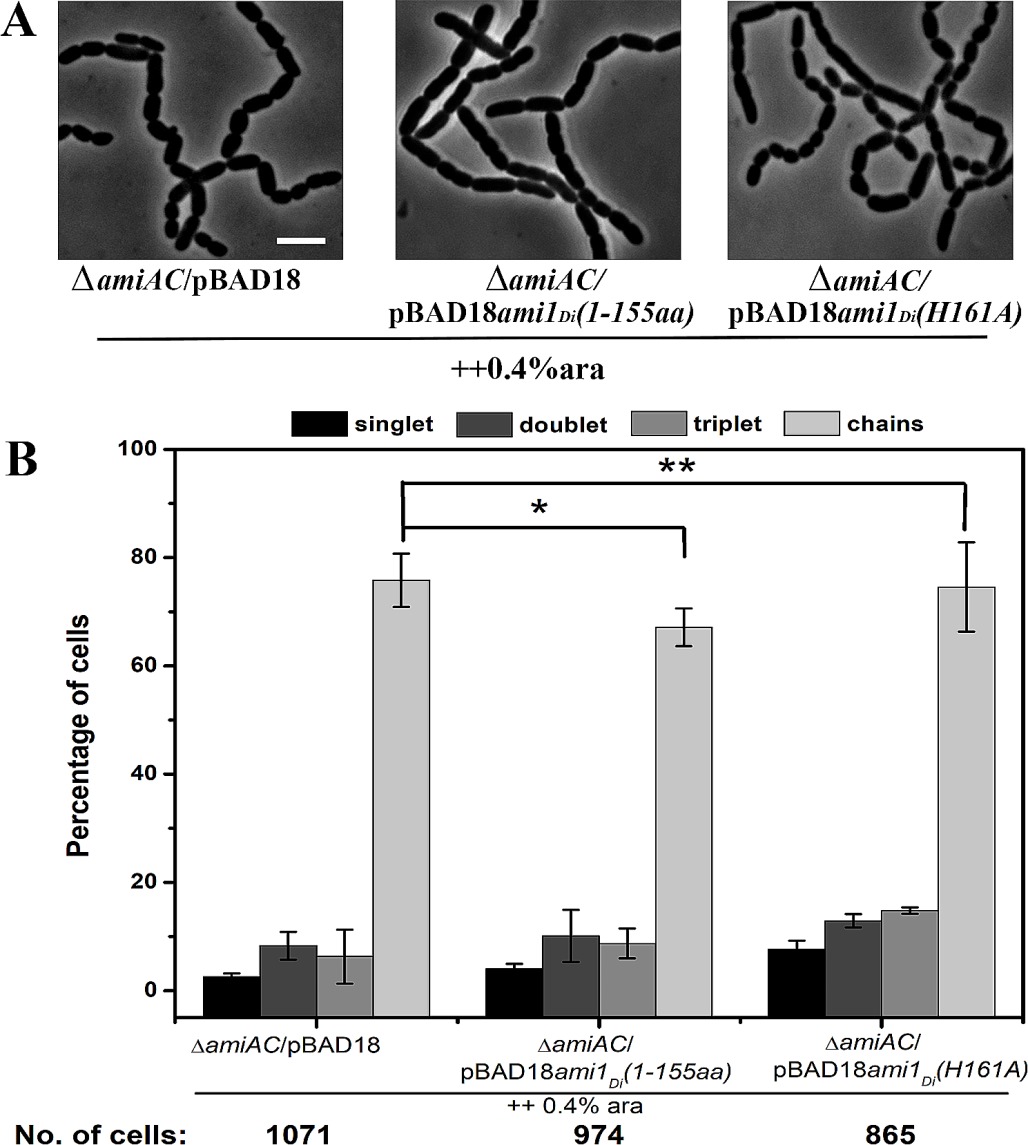
Histidine (H161) is crucial for the lytic activity of *D. indicus* Ami1_*Di*_. (**A**) Phase contrast micrographs showing cells of strains RP21 (MG1655 *ΔamiA::frt ΔamiC::frt* /pBAD18), RP102 (MG1655 *ΔamiA::frt ΔamiC::frt* /pBAD18*ami1*_*Di*_^1–155aa^) and RP103 (MG1655 *ΔamiA::frt ΔamiC::frt* /pBAD18*ami1*_*Di*_^H161A^). Cells were grown to OD_600_ ∼ 0.2 and induced with 0.4% L-arabinose for 6 h, and cells were immobilized on 1XPBS agarose pad and imaged. (**B**) Quantitative analysis of phase contrast micrographs (A). Cells were counted as mentioned in Materials and Methods. The absence of the amidase_3 domain (RP102) or inactivation of catalytic activity of the amidase_3 domain by site-directed mutagenesis (RP103) does not complement the chaining phenotype of the RP21 mutant. Datasets are from three independent experiments, and error bars represent standard deviation. P value = RP21 vs. RP102, *p* > 0.05^*^, RP21 vs. RP103, *p* < 0.05^**^. No. of cells – Total number of cells counted for each strain

Both Ami1_*Di*_ and Ami2_*Di*_ proteins contain a C-terminal zinc-dependent catalytic domain known as the Amidase_3 domain (Fig. 1A). To investigate whether Amidase_3 domain is responsible for the hydrolytic activity of Ami1_*Di*_, a C-terminus truncated version of Ami1_*Di*_ was generated. The C-terminus truncated variant of Ami1_*Di*_ (RP103) was unable to suppress the cell separation defects in *E. coli* amidase mutants (RP21), suggesting that the C-terminal catalytic domain is essential for PG cleavage (Fig. 7B). Histidine at the active site is crucial for both Zn^2+^ binding and catalytic activity in amidases [52, 53]. To investigate whether the active site residue plays a similar role in Ami1_*Di*_, Histidine at 161 position was replaced with Alanine. The H161A active site variant (RP103) was unable to complement cell separation defects in *E. coli* amidase mutants (Fig. 7B). Quantitative analysis revealed only an 8% reduction in cell chains when the C-terminus truncated variant was used in the complementation assay, and this was further reduced to 2% when the active site H161A variant was expressed in *E. coli ∆amiAC* background (Fig. 7C). Taken together our data suggests that similar to other known amidases Histidine (His161) residue at the active site of Ami1_*Di*_ is essential for its catalytic activity.

### Ami1_*Di*_ is a close structural homolog of AmiA_*Ec*_ and displays a similar regulatory domain

**Fig. 8:**
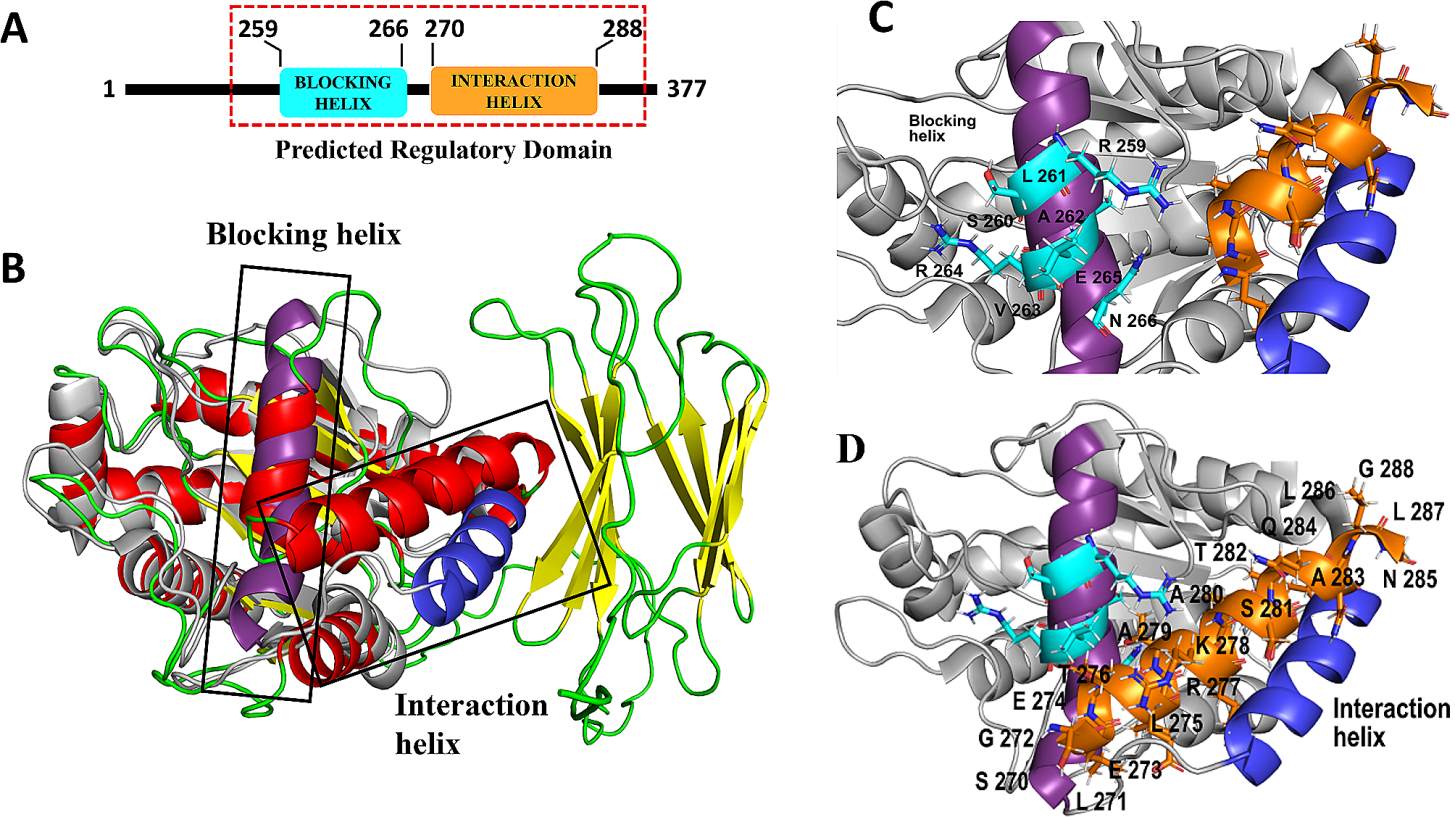
Presence of regulatory domain in Ami1_*Di*_ (**A**) Schematic representation of the predicted regulatory domain of Ami1_*Di*_ comprising blocking helix and interaction helix. (**B**) Cartoon representation of Ami1_*Di*_, colored based on secondary structure (Helices, sheets, and links are color-coded in red, yellow, and green, respectively). This representation is overlaid with AmiA_*Ec*_, displayed in gray. Notably, the blocking helix of AmiA_*Ec*_ is highlighted in violet, while the interacting helix is shown in blue. (**C**) Highlight the corresponding blocking helix in Ami1_*Di*_ is shown in cyan, and the residues are labeled accordingly. The cartoon representation of AmiA is displayed in gray. (**D**) Highlight the corresponding interaction helix in Ami1_*Di*_ is shown in orange, and the residues are labeled accordingly. The Cartoon representation of AmiA_*Ec*_ is shown in gray

The crystal structure of *E. coli* AmiA has been recently studied [54] according to which both AmiA_*Ec*_ and AmiB_*Ec*_ contain regulatory domains that consist of a blocking helix (comprising residues 158–174 in AmiA_*Ec*_ and 294–310 in AmiB_*Ec*_) and an interaction helix (encompassing residues 180–192 in AmiA_*Ec*_ and 320–332 in AmiB_*Ec*_) [54]. The blocking helix functions as an autoinhibitory regulator of AmiA, whereas the interaction helix serves as a binding site for EnvC [55]. We assessed the structural similarity between Ami1_*Di*_ and Ami2_*Di*_ with AmiA (PDB id: 8C2O) and AmiB (PDB id: 8C0J) and computing the RMSD with the cealign command in PyMOL. Our analysis indicates that Ami1_*Di*_ exhibits a closer structural resemblance to AmiA, with an RMSD of 3.25 Å calculated over 216 residues. In contrast, Ami2_*Di*_ shows structural similarity to AmiB, with an RMSD of 2.82 Å calculated over 104 residues. Structural analysis was further extended to predict the regulatory domains and equivalent residues in Ami1_*Di*_ (Fig. 8A & B) compared to AmiA_*Ec*_. Our results predicted the presence of a regulatory domain in Ami1_*Di*_ comprising of a blocking helix (Arg259, Ser260, Leu261, Ala262, Val263, Arg264, Glu265, and Asn266) (Fig. 8C) and an interaction helix (Ser270, Leu271, Gly272, Glu273, Glu274, Leu275, Thr276, Arg277, Lys278, Ala279, Ala280, Ser281, Thr282, Ala283, Gln284, Asn285, Leu286, Leu287, and Gly288) (Fig. 8D). Our results indicate that Ami1_*Di*_ contains a regulatory domain, and its catalytic activity may be regulated comparably to *E. coli* amidases. However, the prediction of equivalent residues in the regulatory domain of Ami2_*Di*_ compared to AmiB_*Ec*_ had lower coverage, primarily because of missing residues.

## Discussion

In this paper, we have characterized the amidases involved in peptidoglycan hydrolysis in *D. indicus*. *D. indicus* genome encodes two amidases, Ami1_*Di*_ and Ami2_*Di*_. Both enzymes are able to supress cell separation defects in *E. coli* amidase mutants, indicating that Ami1_*Di*_ and Ami2_*Di*_ may play a role in septal peptidoglycan splitting. Most *N*-acetylmuramyl-l-alanine amidases fall into 3 families: NALAA-2, NALAA-3 and NALAA-5 [18]. Members of the Amidase_3 family are zinc-dependent enzymes and include bacterial and phage amidases. Ami1_*Di*_ and Ami2_*Di*_ contain Amidase_3 catalytic domain at C-terminus, and comparative structural analysis identified the conserved active site motif –GHGG- in both amidases (Figs. 1A and 5). In Ami1_*Di*_, the active site residues comprised of G160, H161, G162, and G163 of these H161 is involved in zinc binding too (Fig. 5). Zn^2+^ binding is essential for catalytic activity, and the H161A active site variant of Ami1_*Di*_ enzyme did not display PG splitting activity in *E. coli* (Fig. 7). The N-terminus is predicted to have signal peptide sequences (Fig. 1A). However, both proteins lack cell wall binding domains (CBD) and AMIN domain at N-terminus. The absence of CBD has also been observed in AmiC from *C. crescentus* [30] and *M. tuberculosis* amidase Rv3717 [56].

Preserving the integrity of the cell wall at all times is paramount for bacterial viability, and peptidoglycan cleavage activity of amidases is tightly controlled. In *E. coli*, amidase activation requires direct contact with LytM domain-containing protein EnvC and NlpD [11]. NlpD specifically activates AmiC, while EnvC can activate both AmiA and AmiB. Recent studies revealed that activators NlpD and EnvC interact with their cognate amidases, displace the auto-inhibitory helix from the amidases’ active site, and stimulate peptidoglycan hydrolase activity [23, 25, 54]. The crystal structure of *E. coli* AmiA enzyme has a regulatory domain that consists of a blocking helix and an interaction helix [54]. The blocking helix is involved in auto-inhibition and occludes the zinc active site, and the interaction helix mediates binding with activator EnvC [54]. In our study, the AlphaFold2 structure of Ami1_*Di*_ displayed a high resemblance to *E. coli* AmiA. Ami1_*Di*_ is also predicted to have a regulatory domain consisting of both a blocking helix (259–266 residues) and an interaction helix (278–288 residues) (Fig. 8). This prompted us to predict that Ami1_*Di*_ may also be auto-inhibited and require interaction with an activator protein to stimulate PG cleavage. *D. indicus* genome mining revealed the presence of amidase activators LysM domain-containing peptidoglycan endopeptidase NlpC/P60 (WP_088249103) and M23 family metallopeptidase EnvC (WP_229843994). EnvC_*Di*_ has high sequence similarity with the *E. coli* homolog (31.4%) and also has the conserved metal-binding sites of the peptidase_M23 domain (Fig. S5). Taken together, our data suggest that the amidase/cognate circuit model may be operational in *D. indicus*.

AlphaFold2 structure of Ami2_*Di*_ protein revealed some significant differences compared to Ami1_*Di*_. Ami2_*Di*_ was predicted to be a three-domain protein, comprising of a β-sandwich N-terminal domain, an α/β C-terminal domain, and an additional β-sandwich domain situated between the two primary domains (Fig. 5D). Moreover, overexpression of Ami2_*Di*_ in *E. coli* increased cell lysis, indicating that these enzymes may play functionally distinct roles in *D. indicus.* Both *Deinococcus* and *Thermus* genera are extremophiles known to survive harsh environmental conditions [57]. The Deinococcaceae family has a unique cell envelope attributed to their survival in extreme environments. A representative member of this family, *D. radiodurans*, while stains gram-positive, has an envelope architecture of Gram-negative bacteria [58, 59]. *D. radiodurans* cell envelope consists of an inner membrane, a peptidoglycan layer, and an outer membrane [60–63]. However, the outer membrane lacks classical lipopolysaccharides present in Gram-negative bacteria [64, 65]. *Deinococcus* also has a unique cell wall consisting of ornithine-Gly-peptidoglycan and differs from mDAP-peptidoglycan in *E. coli* [66, 67]. Both Ami1_*Di*_ and Ami2_*Di*_ could cleave *D. indicus* cell wall, indicating that both enzymes can hydrolyze ornithine-Gly-peptidoglycan. However, these enzymes may not be specific for ornithine-containing peptidoglycan as both enzymes were active in *E. coli* cells, too. Our study is the first to characterize cell wall amidases from *Deinococcus indicus* DR1 and future investigations would help to understand their role in the biogenesis and division of complex cell envelopes of these bacteria.

### Electronic supplementary material

Below is the link to the electronic supplementary material.

Supplementary Material 1

Supplementary Material 2

## Data Availability

The coordinates of Ami1 (https://www.modelarchive.org/doi/10.5452/ma-ot0l4) and Ami2 (https://www.modelarchive.org/doi/10.5452/ma-55yq9) have been deposited in ModelArchive. The rest of the data is provided within the manuscript or supplementary files.
