# Supplementary material for: Structure predictions and functional insights into Amidase_3 domain containing N-acetylmuramyl-L-alanine amidases from Deinococcus indicus DR1
Source: BMC Microbiol. 2024 Mar 26;24:101. doi: 10.1186/s12866-024-03225-4 (PMC10964502; doi:10.1186/s12866-024-03225-4)
Supplement: Supplementary file 1 — Supplementary Material 1 [file 12866_2024_3225_MOESM1_ESM.docx]

**Supplementary file**

**Structure predictions and functional insights into Amidase_3 domain containing *N*-acetylmuramyl-L-alanine amidases from *Deinococcus indicus* DR1**

**Malvika Modi^1^, Menaka Thambiraja^2^, Archana Cherukat^1¶^, Ragothaman M Yennamalli^2^ and Richa Priyadarshini^1*^**

^1^ Department of Life Sciences, School of Natural Sciences, Shiv Nadar Institution of Eminence, Gautam Buddha Nagar, Uttar Pradesh, 201314, India

^2^ Department of Bioinformatics, School of Chemical and Biotechnology, SASTRA Deemed to be University, Thanjavur, Tamil Nadu, 613401, India

***** Corresponding author

**Correspondence and reprints**:

**Dr. Richa Priyadarshini**

Department of Life Sciences, School of Natural Sciences,

Shiv Nadar Institution of Eminence

Gautam Buddha Nagar, Uttar Pradesh, India.

Ph no: +91-120-3819100 Ext. 220

Email: [richa.priyadarshini@snu.edu.in](mailto:richa.priyadarshini@snu.edu.in) (RP)

¶ Present Address:

Department of Biology, Graduate School of Arts and Sciences

Wake Forest University

1834 Wake Forest Rd, Winston-Salem, USA

**Supplementary file**


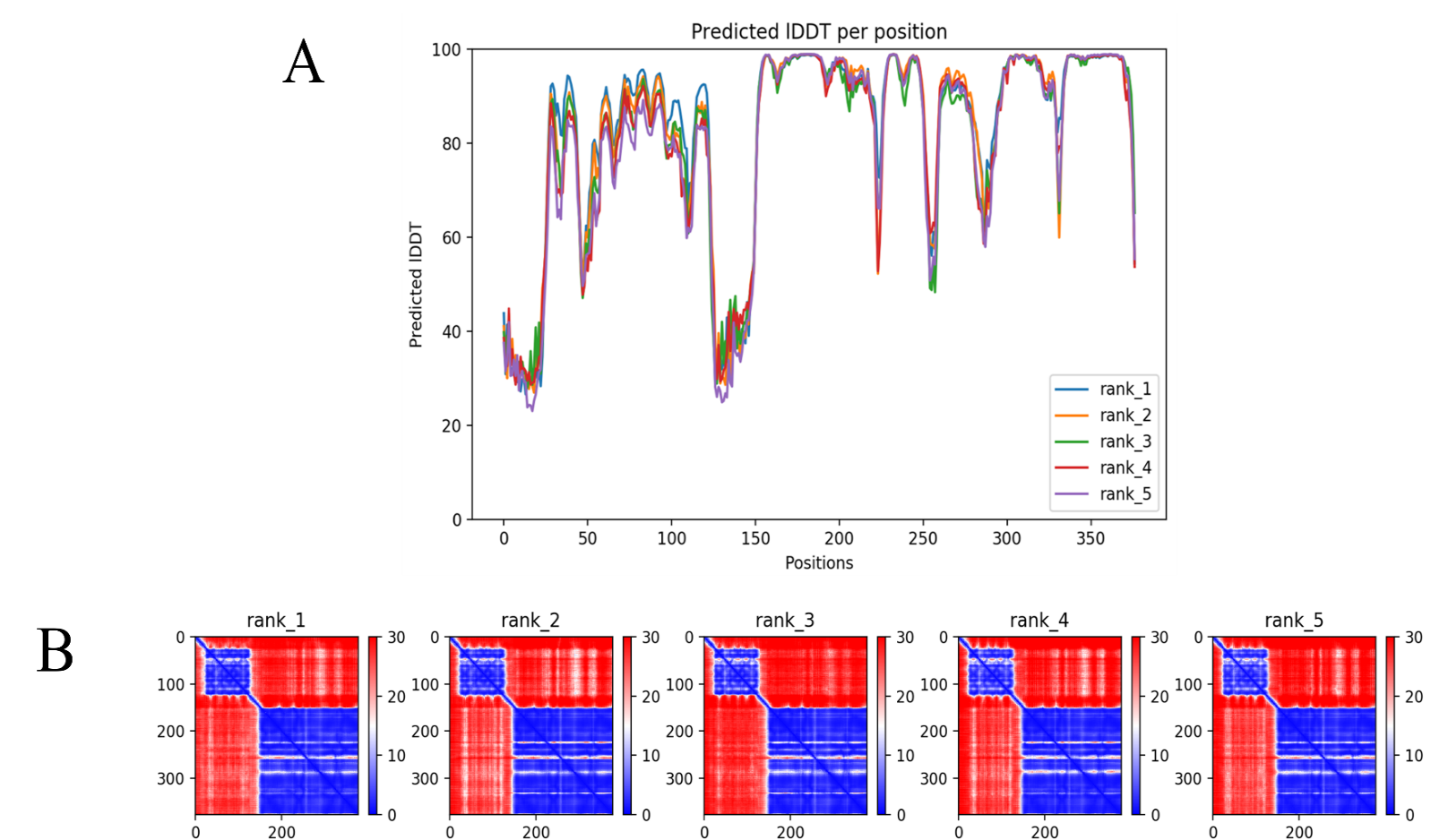


**Supplementary Figure 1**: (a) pLDDT score for each residue in the Ami1*_Di_* structure predicted by AlphaFold2 (b) PAE plots for the five models predicted by AlphaFold2.


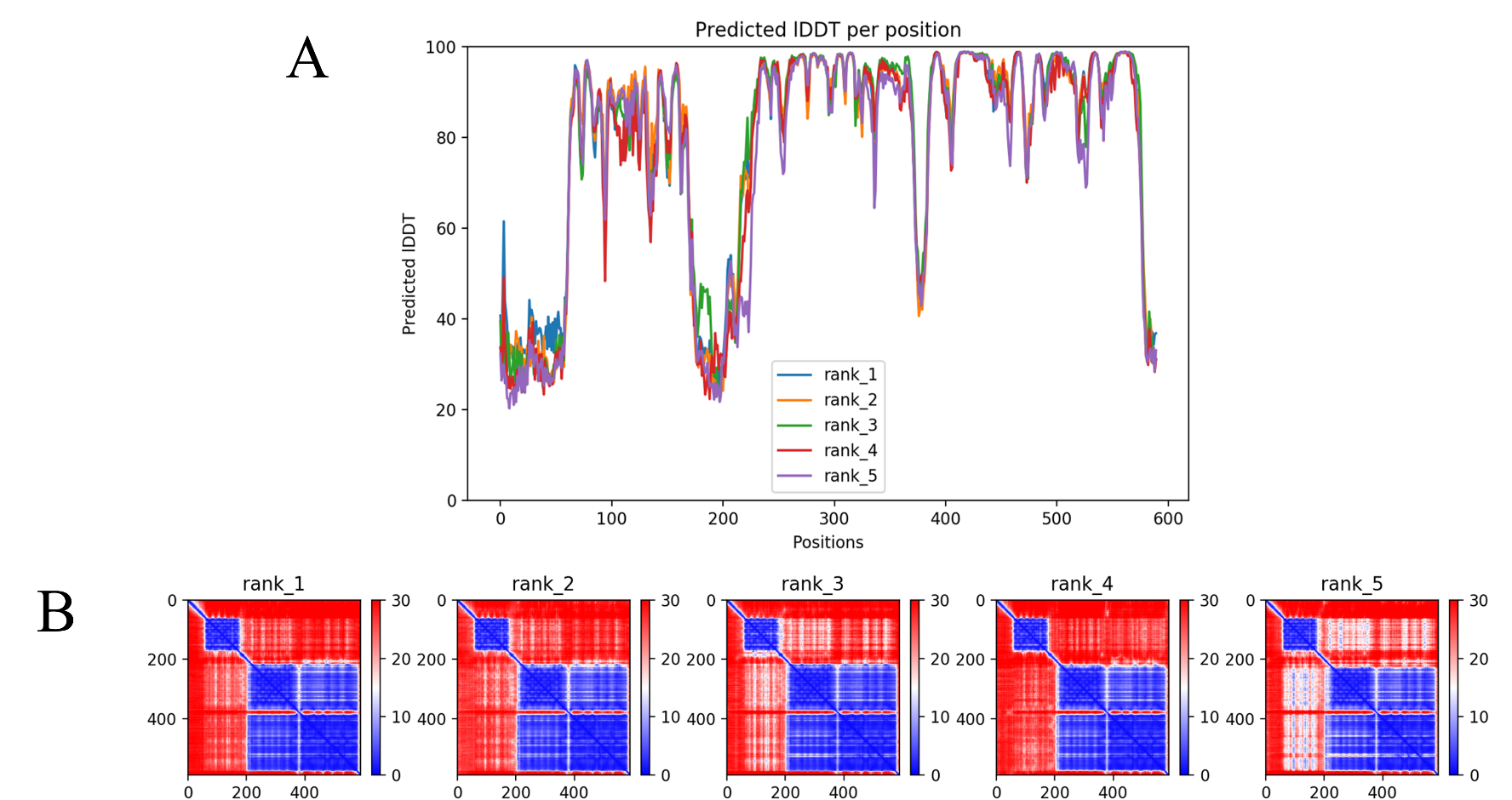


**Supplementary Figure 2:** (a) pLDDT score for each residue in the Ami2*_Di_* structure predicted by AlphaFold2 (b) PAE plots for the five models predicted by AlphaFold2.


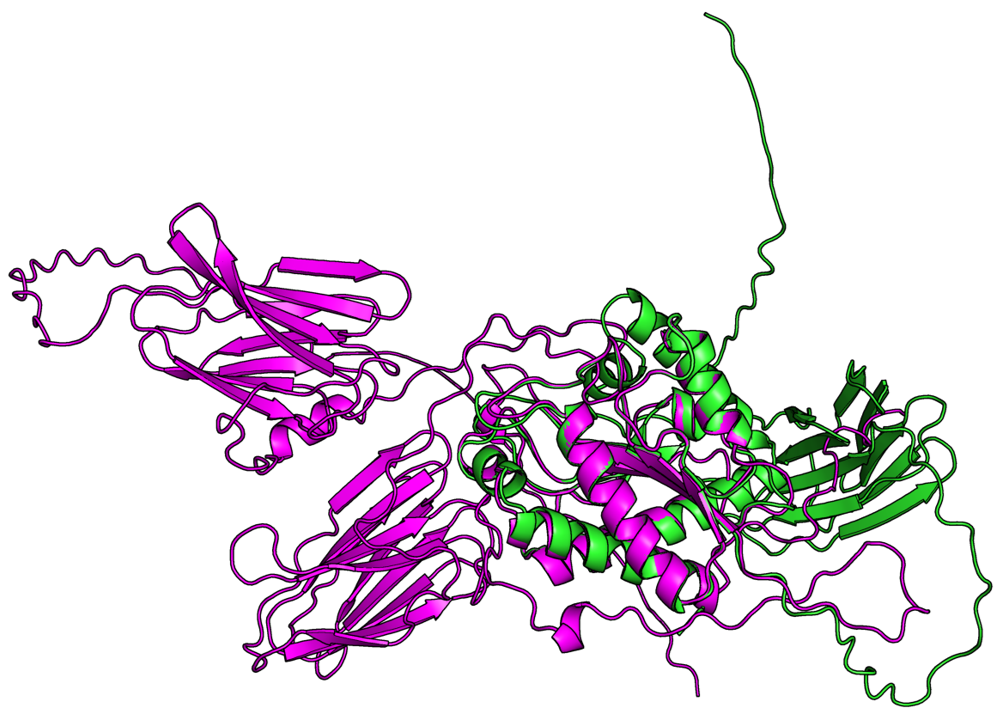


**Supplementary Figure 3 :** Superposed structures of Ami1*_Di_* (colored green) and Ami2*_Di_* (colored magenta) showing the alpha/beta domain showing high structural similarity.


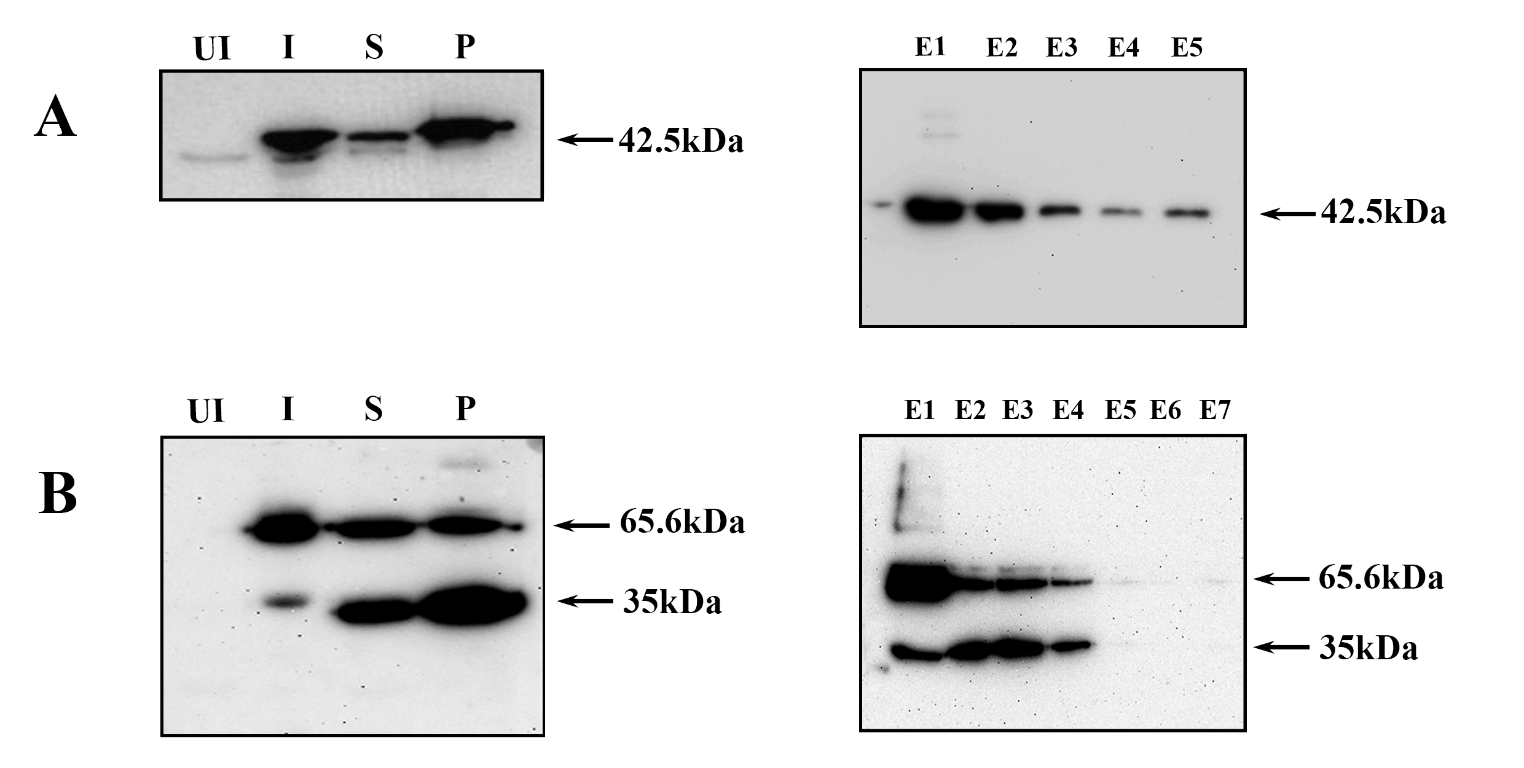


**Supplementary Figure 4** – (A) Western Blot of induced fraction and purified protein elutes of Ami1*_Di_* (̴ 42.5kDa) and (B) Ami2*_Di_* (̴ 65.6kDa). RP105 and RP106 were grown till OD_600_ ̴ 0.6 and induced with 0.5 mM IPTG for 4 h at 25 °C. Supernatant and pellet fractions were separated after sonication. uninduced (UI), induced (I), supernatant (S) and pellet (P) fraction (E) elutes were loaded on SDS-PAGE gel and Western blot was performed with anti-His tag monoclonal antibody.


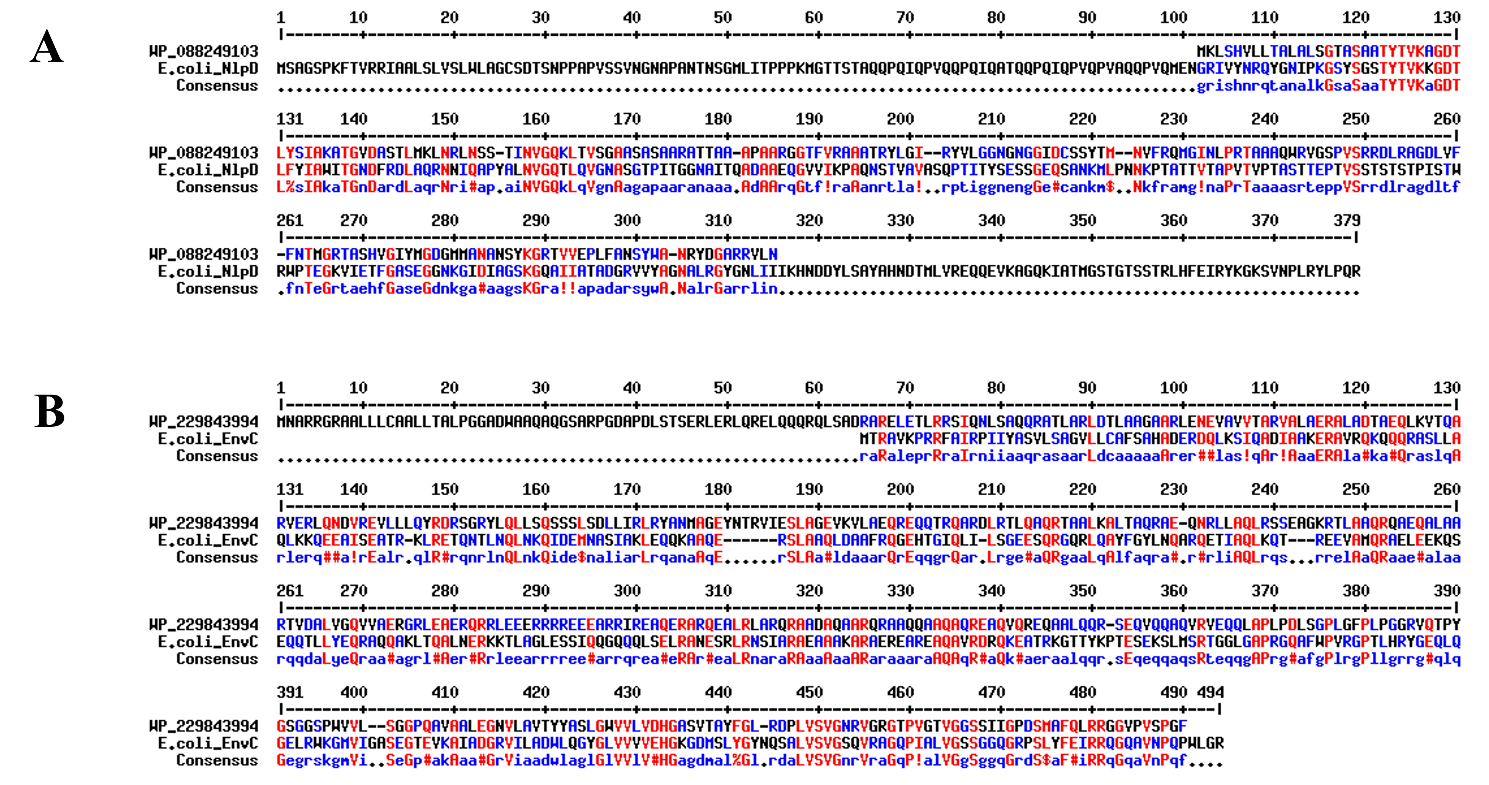


**Supplementary figure 5**

Similarity between NlpC/P60 (WP_088249103) and EnvC (WP_229843994) of *D. indicus* and NlpD and EnvC of *E. coli*

(A) Multiple Sequence Alignment of *D. indicus* NlpC/P60 (WP_088249103) sequence and *E. coli* NlpD in MULTALIN. (B) Multiple Sequence Alignment of *D. indicus* EnvC (WP_229843994) sequence and *E. coli* EnvC in MULTALIN. Highly conserved residues are represented by red color, weakly conserved residues by blue whereas a dot in the consensus line represents no conserved residue. The conserved active site residues is boxed in black.

**Supplementary Table 1**: Structural homologs of Ami1*_Di_* and Ami2*_Di_* from DALI server with hits having a Z-score of 10 and above.

|  | Ami1*_Di_* structural homologs | | Ami2*_Di_* structural homologs | |
| --- | --- | --- | --- | --- |
| 1. No | PDB Id | Z-score | PDB Id | Z-score |
| 1. | 1JWQ | 28.9 | 4RN7 | 28.3 |
| 2. | 5EMI | 28.8 | 5EMI | 27.8 |
| 3. | 4RN7 | 28.3 | 1JWQ | 27.5 |
| 4. | 5J72 | 27.6 | 5J72 | 25.9 |
| 5. | 4LQ6 | 25.8 | 4LQ6 | 24.4 |
| 6. | 4BIN | 25.5 | 4M6G | 24.0 |
| 7. | 4M6G | 25.3 | 4BIN | 22.9 |
| 8. | 3NE8 | 24.8 | 3NE8 | 22.7 |
| 9. | 3CZX | 22.3 | 3QAY | 20.7 |
| 10. | 3QAY | 22.1 | 1XOV | 19.7 |
| 11. | 1XOV | 20.4 | 4OKO | 12.5 |
| 12. | 3A9L | 12.2 | 2GFQ | 11.3 |
| 13. | 4OKO | 12.1 |  |  |
| 14. | 2GFQ | 11.1 |  |  |

**Supplementary table 2** – Primers used in this study

| Primers used in this study | | |  |
| --- | --- | --- | --- |
|  | **Primer name** | **Primer Sequence** | **Remarks** |
| 1 | pBAD*ami1_Di_* FP | CCCCCGCTAGCGAGGAGGAAGTCATCGTGAAGCTGCCATCTCTCCTG | Amplification of full-length *ami1_Di_* for cloning in pBAD18 vector |
| 2 | pBAD*ami1_Di_* RP | CCCCCGGTACCTCACTTGGTGTGCAGGAAGTC |  |
| 3 | pBAD*ami2_Di_* FP | CCCCCGCTAGCGAGGAGGAAGTCATCATGAAGCTGCCTGTCA | Amplification of full-length *ami2_Di_* for cloning in pBAD18 vector |
| 4 | pBAD*ami2_Di_* RP | CCCCCGGTACCTCAGCGGGAGGTGCCG |  |
| 5 | pBAD*ami1_Di_*Tn (1-155aa) FP | CCCCCGCTAGCGAGGAGGAAGTCATCGTGAAGCTGCCATCTCTCCTG | Amplification of truncated (1-155aa) *ami1_Di_* for cloning in pBAD18 vector |
| 6 | pBAD*ami1_Di_*Tn (1-155aa) RP | CCCCCGGTACCTCACACGCGCGGCCGGGTGGG |  |
| 7 | pBAD*ami1_Di_*SDM (H161A) FP | CTCGATCCCGGTGCCGGCGGCACC | Amplification of active site mutant (H161A) *ami1_Di_* for cloning in pBAD18 vector |
| 8 | pBAD*ami1_Di_*SDM (H161A) FP | GTCGGTGCCGCCGGCACCGGGATC |  |
| 9 | pET28b*ami1_Di_* FP | CCCCCGAATTCGTGAAGCTGCCATCTCTCCT | Amplification of full-length *ami1_Di_* – 6X His-Tag for cloning in pET28b vector |
| 10 | pET28b*ami1_Di_* RP | CCCCCAAGCTTTCAATGATGATGATGATGATGTCACTTGGTGTGCAGGAAG |  |
| 11 | pET28b*ami2_Di_* FP | CCCCCGAATTCATGAAGCTGCCTGTCATC | Amplification of full-length *ami2_Di_* – 6X His-Tag for cloning in pET28b vector |
| 12 | pET28b*ami2_Di_* RP | CCCCCAAGCTTTCAATGATGATGATGATGATGGCGGGAGGTGCCGGC |  |
