# Supplementary material for: Structure predictions and functional insights into Amidase_3 domain containing N-acetylmuramyl-L-alanine amidases from Deinococcus indicus DR1
Source: BMC Microbiol. 2024 Mar 26;24:101. doi: 10.1186/s12866-024-03225-4 (PMC10964502; doi:10.1186/s12866-024-03225-4)
Supplement: Supplementary file 2 — Supplementary Material 2 [file 12866_2024_3225_MOESM2_ESM.docx]

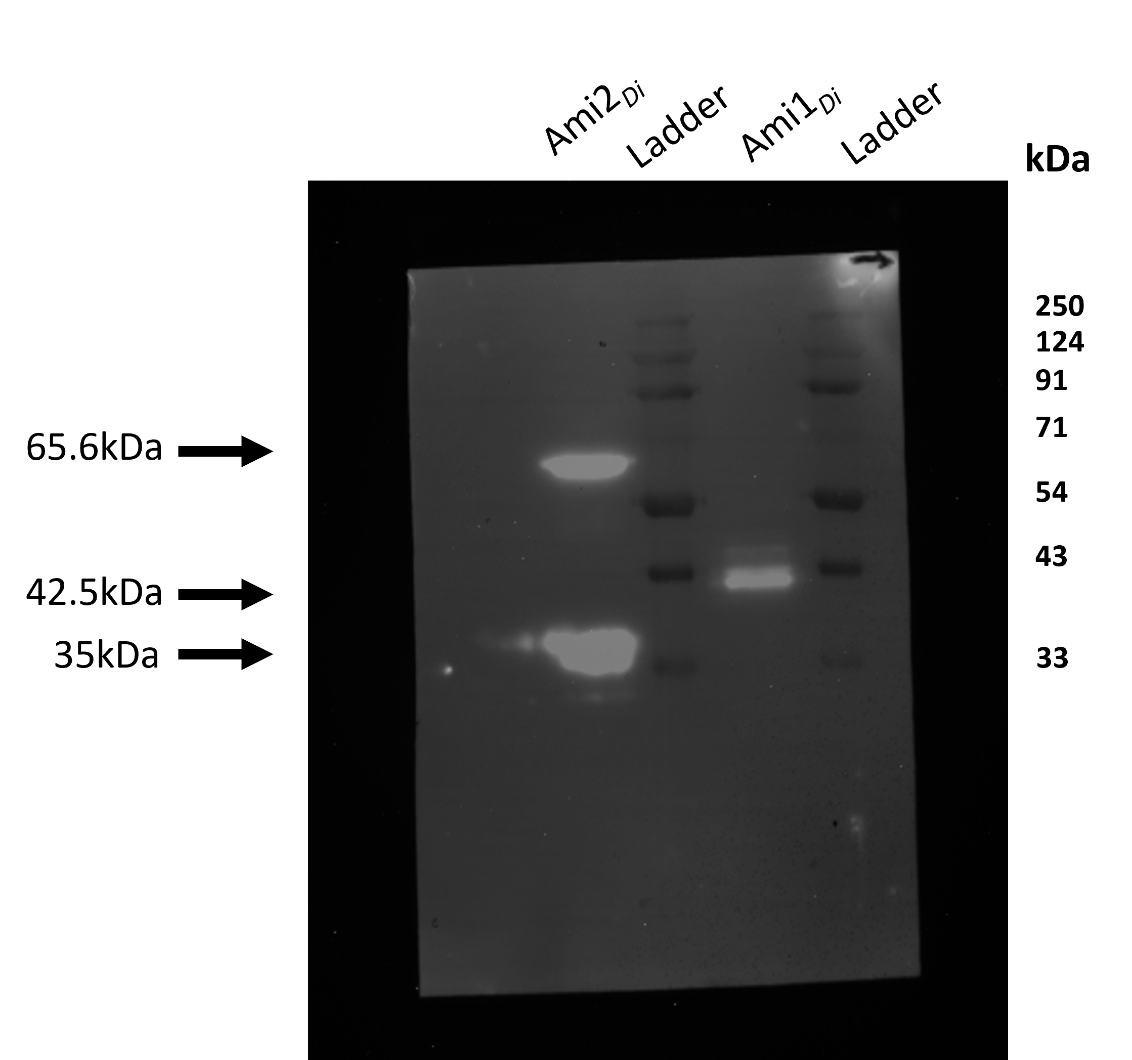


**Additional Figure 1 (Fig.6A)** - Western blot showing purified His-tagged Ami2*_Di_* (65.6kDa) – Lane 1 and Ami1*_Di_* (42.5kDa) – Lane 2 protein heterologously produced in *E.coli* BL21 (RP106 & RP105).


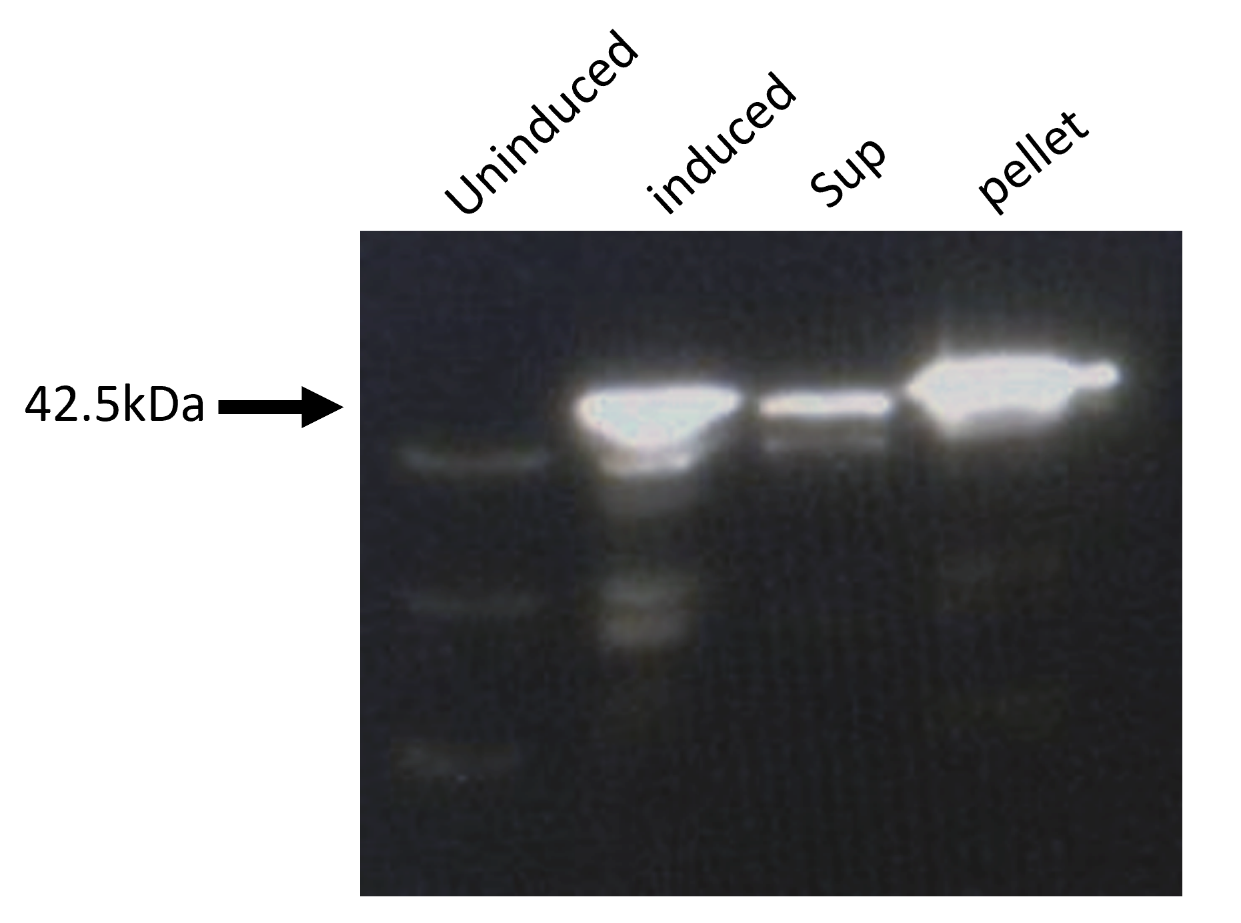


**Additional Figure 2 (Supplementary figure. 4A (i))** – Western blot of induced fraction of strain RP105. Lanes showing expression of His-tag in uninduced, induced, supernatant and pellet fractions, respectively of Ami1*_Di_*.


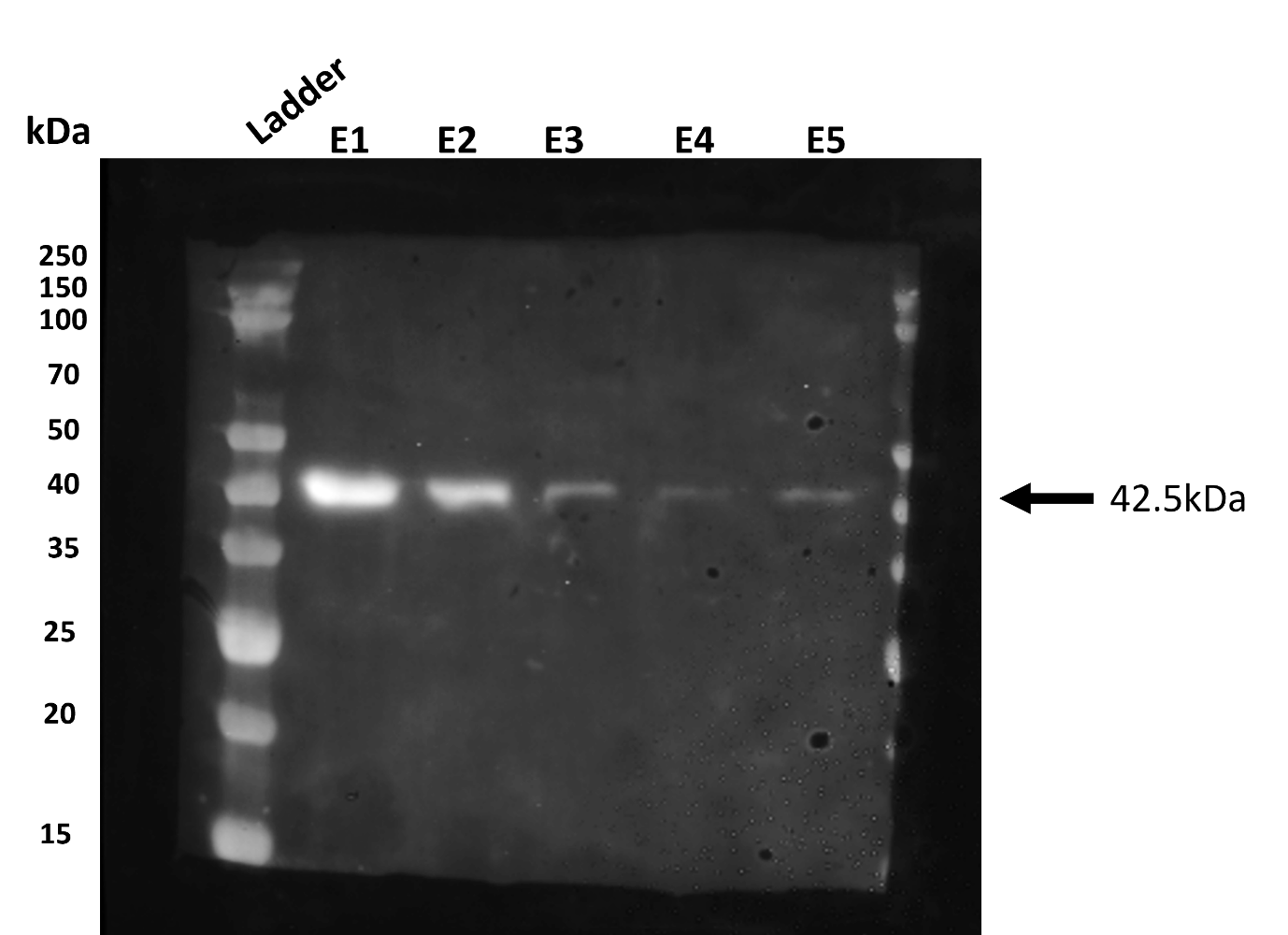


**Additional Figure 3 (Supplementary figure. 4A (ii)** – Western Blot of purified protein elutes (E1, E2, E3, E4, E5) of Ami1*_Di_* (approx. 42.5kDa).


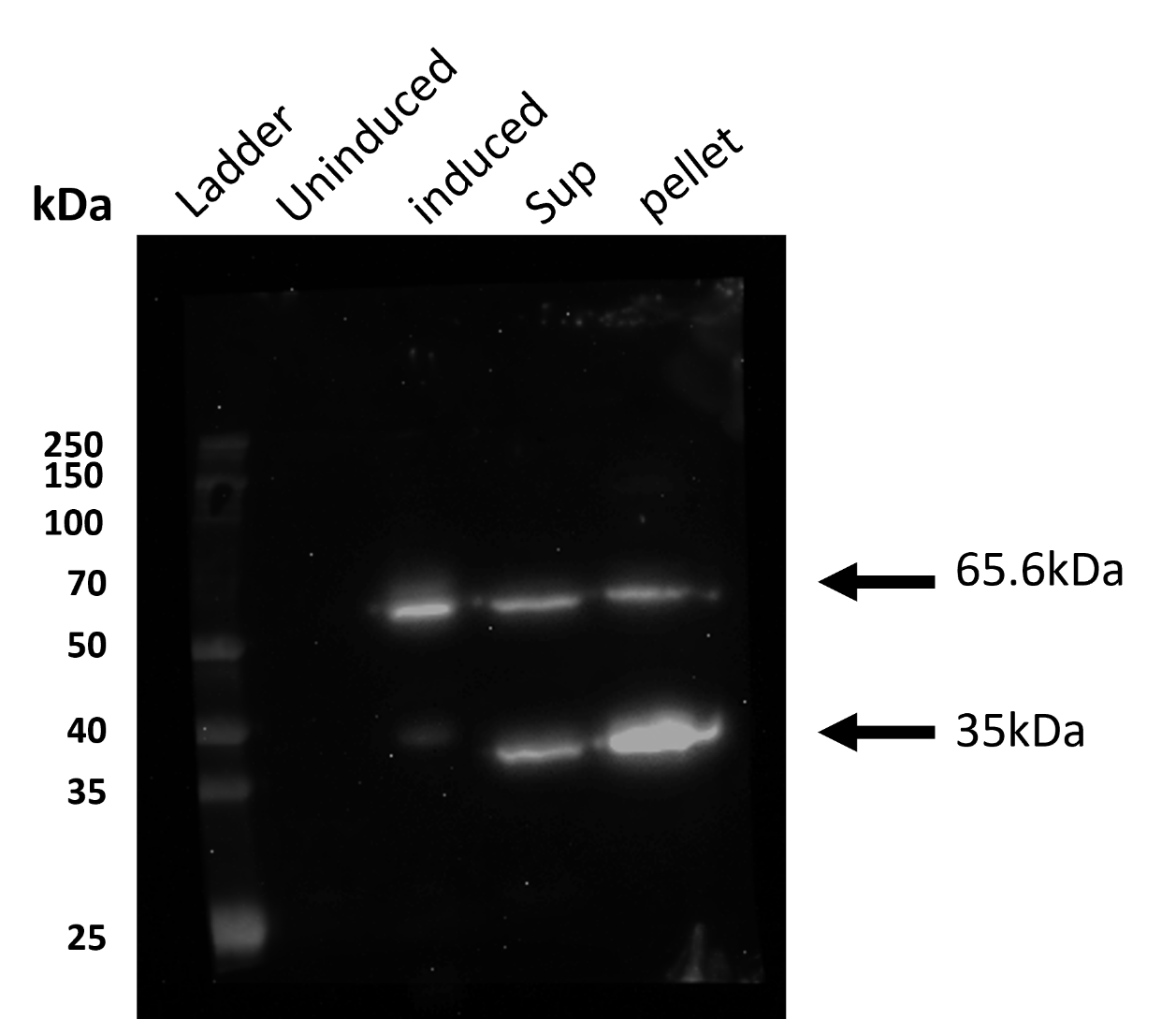


**Additional figure 4 (Supplementary Figure 4B (i)**) - Western blot of induced fraction of strain RP105. Lanes showing expression of His-tag in uninduced, induced, supernatant and pellet fractions, respectively of Ami2*_Di_*.


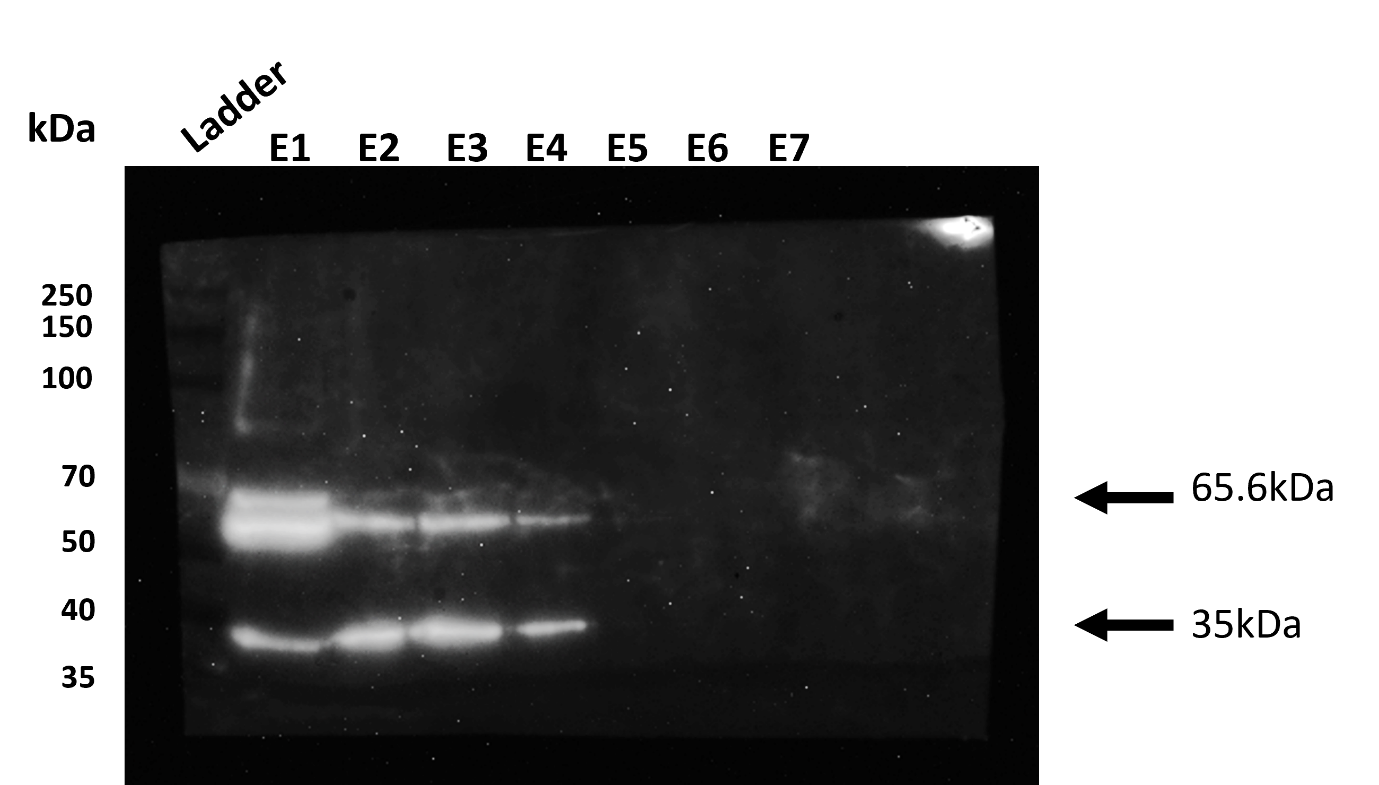


**Additional Figure 5 (Supplementary figure. 4A (ii)** – Western Blot of purified protein elutes (E1, E2, E3, E4, E5, E6, E7) of Ami2*_Di_* (approx. 42.5kDa).
